# Translesion activity of PrimPol on DNA with cisplatin and DNA–protein cross-links

**DOI:** 10.1038/s41598-021-96692-y

**Published:** 2021-09-02

**Authors:** Elizaveta O. Boldinova, Anna V. Yudkina, Evgeniy S. Shilkin, Diana I. Gagarinskaya, Andrey G. Baranovskiy, Tahir H. Tahirov, Dmitry O. Zharkov, Alena V. Makarova

**Affiliations:** 1 Institute of Molecular Genetics, National Research Center «Kurchatov Institute», Kurchatov sq. 2, Moscow, Russia 123182; 2 Eppley Institute for Research in Cancer and Allied Diseases, Fred & Pamela Buffett Cancer Center, University of Nebraska Medical Center, Omaha, NE 68198 USA; 3 Institute of Chemical Biology and Fundamental Medicine, Siberian Branch of the Russian Academy of Sciences, 8 Lavrentiev Avenue, Novosibirsk, Russia 630090; 4 Novosibirsk State University, 2 Pirogova St., Novosibirsk, Russia 630090

**Keywords:** Biochemistry, Molecular biology

## Abstract

Human PrimPol belongs to the archaeo-eukaryotic primase superfamily of primases and is involved in de novo DNA synthesis downstream of blocking DNA lesions and non-B DNA structures. PrimPol possesses both DNA/RNA primase and DNA polymerase activities, and also bypasses a number of DNA lesions in vitro. In this work, we have analyzed translesion synthesis activity of PrimPol in vitro on DNA with an 1,2-intrastrand cisplatin cross-link (1,2-GG CisPt CL) or a model DNA–protein cross-link (DpCL). PrimPol was capable of the 1,2-GG CisPt CL bypass in the presence of Mn^2+^ ions and preferentially incorporated two complementary dCMPs opposite the lesion. Nucleotide incorporation was stimulated by PolDIP2, and yeast Pol ζ efficiently extended from the nucleotides inserted opposite the 1,2-GG CisPt CL in vitro. DpCLs significantly blocked the DNA polymerase activity and strand displacement synthesis of PrimPol. However, PrimPol was able to reach the DpCL site in single strand template DNA in the presence of both Mg^2+^ and Mn^2+^ ions despite the presence of the bulky protein obstacle.

## Introduction

Correct DNA replication and cell cycle progression requires coordinated work of many enzymes and auxiliary proteins. Human PrimPol is a primase belonging to the archaeo-eukaryotic primase (AEP) superfamily, which possesses both DNA/RNA primase and DNA polymerase activities^1,2^. Emerging evidence suggests the importance of PrimPol role in genome stability maintenance. PrimPol is involved in de novo DNA synthesis downstream of DNA lesions and non-B DNA structures blocking the replication machinery in nuclei and mitochondria^3–7^. PrimPol-dependent reinitiation of DNA synthesis interplays with other DNA damage tolerance pathways such as fork reversal and homologous recombination^8,9^.

Besides its primase activity, PrimPol is capable of bypassing some DNA lesions in vitro suggesting that PrimPol may perform DNA translesion synthesis (TLS). PrimPol efficiently bypasses 8-oxoguanine (8-oxoG), 5-formyluracil, *O*^6^-methylguanine and abasic site in the presence of Mg^2+^ ions^1,2,10,11^. In Mn^2+^-catalyzed reactions, PrimPol synthesizes through additional blocking lesions such as 1,*N*^6^-ethenoadenine and thymine glycol^10^. PrimPol also bypasses UV-induced lesions: *cis-syn* T–T dimers (CPD) and T–T (6–4) photoproducts^2,5,12^. It was suggested that PrimPol can “skip” blocking DNA lesions rather than incorporate dNMPs opposite these DNA adducts (so-called “pseudo-TLS” or the template “scrunching” mechanism). It is believed that the primase activity is the primary function of PrimPol in cells^4–6^ but, if a complex damage is present, the situation is possible when during de novo DNA synthesis PrimPol encounters a DNA lesion, and the TLS activity of PrimPol might contribute to the DNA damage tolerance. PrimPol binding to DNA and its activity can be regulated by accessory proteins: replication protein A (RPA)^13–15^ and DNA polymerase δ-interacting protein 2 (PolDIP2)^16^.

DNA cross-links (CL) represent an abundant class of DNA lesions in a genome. They may be formed between bases of one DNA strand (intrastrand CL), bases of different DNA strands (interstrand CL) and between DNA and a protein molecule (DNA–protein CL, DpCL). Among crosslinking agents, cisplatin represents a special interest due to its significance as an antitumor chemotherapy drug^17^. The most common cisplatin-induced Pt–DNA adducts are 1,2-intrastrand CLs between adjacent guanine bases (1,2-GG CisPt CL) (60–65%) and adjacent adenine and guanine bases (20–25%). The remaining 10–20% include monoadducts, 1,3- and 1,4-intrastrand CLs between purines separated by one or two nucleotides, interstrand CLs and DpCLs (reviewed in^18^). Cisplatin adducts represent a block for replicative polymerases^19^. Cisplatin CLs halt DNA replication and induce an initial transient S-phase arrest, which is followed by a persistent G2/M arrest and apoptosis^20–22^. On the other hand, efficient repair, TLS and replication restart might contribute to tumor resistance to cisplatin (reviewed in^23^).

DpCLs can be formed by chemotherapeutic drugs such as cisplatin and some environmental agents (formaldehyde, 1,3-butadiene, hexavalent chromium). DpCLs also can arise from reactions of radicals with DNA, from abortive enzymatic reactions of topoisomerases or generation of AP sites (reviewed in^24^). Moreover, several DNA repair proteins can be covalently trapped during base excision repair. DNA-glycosylases (such as Nth, Fpg, NEIL1) and other enzymes with associated lyase activity (such as Pol β, Pol λ or PARP1) may become irreversibly trapped by oxidized or even regular AP sites^25–31^. DpCLs as extremely bulky adducts interfere replication and transcription with lethal consequences if unrepaired^32–34^. Removal of a DpCL from DNA is challenging due to bulkiness of the adducts which interfere sterically with the assembly of the mammalian NER complex^35^. One of proposed pathways for a DpCL removal is proteolytic degradation followed by TLS over a DNA-peptide CL^36,37^.

The DNA polymerase activity of PrimPol on DNA with cisplatin and DNA–protein CLs is yet to be investigated. In this work, we analyzed the ability of PrimPol to replicate past an 1,2-GG CisPt CL and a model DNA–protein crosslinks in vitro. We demonstrated that PrimPol is capable of bypass of the cisplatin CL in the presence of Mn^2+^ ions and PolDIP2 with low efficiency. Pol ζ efficiently extended the synthesis beyond the lesion. The synergetic effect of PrimPol, PolDIP2 and Pol ζ on DNA with the 1,2-GG CisPt CL was more efficient compared to Pol κ. We also showed that PrimPol is unable to bypass a DpCL neither in the template strand of double-stranded DNA nor in the displaced strand of double-stranded DNA. However, if the substrate contains DpCL in the single-stranded template DNA, PrimPol is able to reach the cross-link site despite the presence of the very bulky protein obstacle. Low yet reproducible primer elongation beyond DpCL was observed in the presence of Mn^2+^ ions.

## Results

### Mn^2+^ ions stimulate bypass of a 1,2-GG cisplatin CL by PrimPol

**Figure 1:**
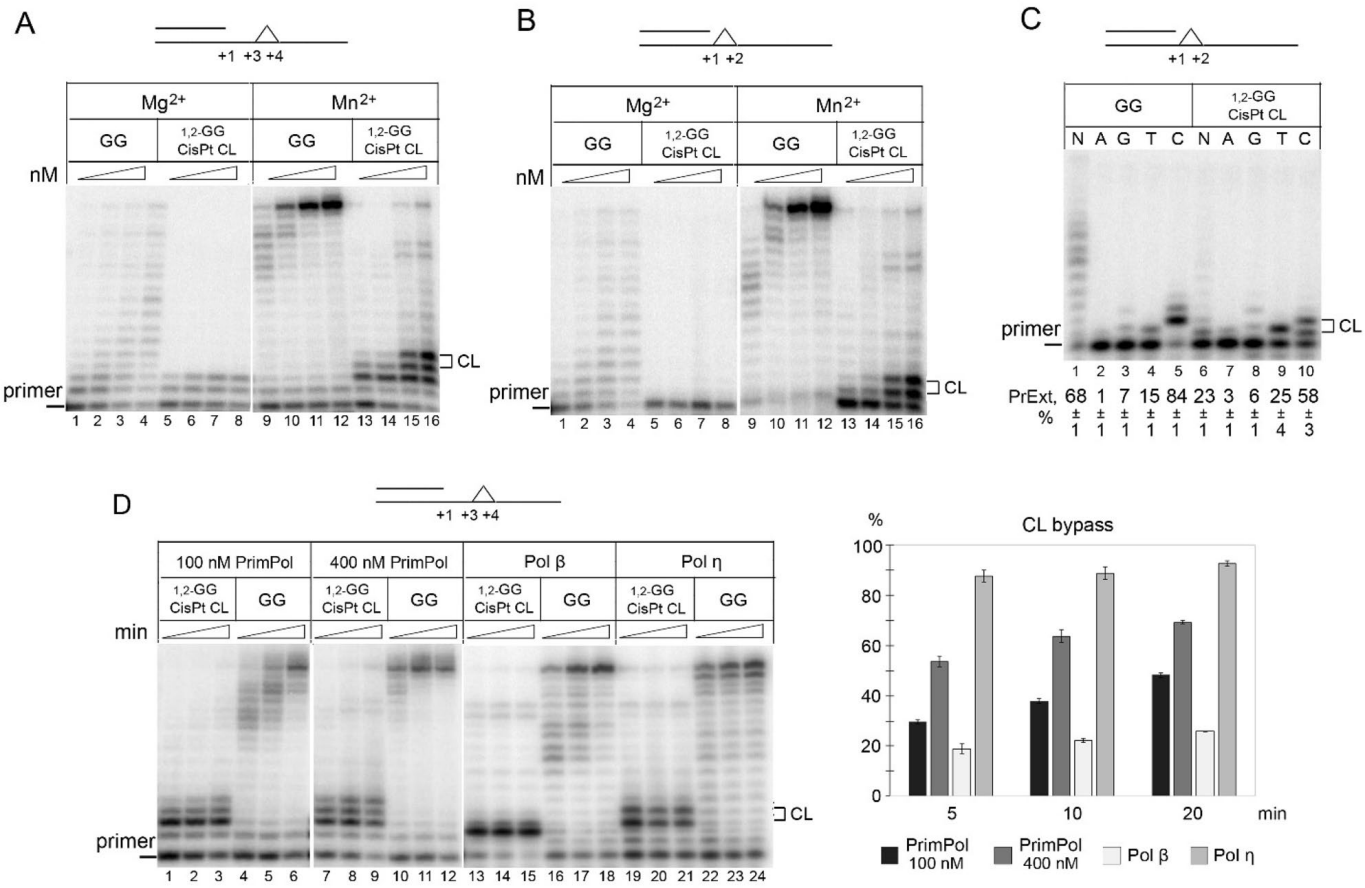
The TLS activity of PrimPol on DNA substrates with the 1,2-GG CisPt CL. (**A**) The TLS activity of PrimPol on DNA substrates containing the 1,2-GG CisPt CL or undamaged GG at the + 3–4 positions downstream of the primer. Reactions were carried out in the presence of 100 – 600 nM PrimPol, 10 mM MgCl_2_ or 0.5 mM MnCl_2_ for 10 min. (**B**) The TLS activity of PrimPol on DNA substrates with the 1,2-intrastrand cisplatin CL or undamaged GG at the + 1–2 position downstream of the primer. Reactions were carried out in the presence of 100 – 600 nM PrimPol, 10 mM MgCl_2_ or 0.5 mM MnCl_2_ for 10 min. (**C**) dNMPs incorporation on DNA substrate with the 1,2-GG CisPt CL or undamaged GG at the + 1–2 position. Reactions were carried out in the presence of 200 nM PrimPol, 0.5 mM MnCl_2_ for 5 min. Experiments were repeated 3 times. The mean values of the primer extension percentages (PrExt) and the standard errors are indicated. (**D**) The TLS activity of PrimPol, Pol β and Pol η on DNA substrate with the 1,2-GG CisPt CL or undamaged GG at the + 3–4 position downstream of the primer. Reactions were carried out in the presence of 0.5 mM MnCl2 (PrimPol) or 10 mM MgCl2 (Pol β and Pol η) for 5, 10 or 20 min. The level of the DNA polymerase activity of Pol β (20 nM), Pol η (10 nM) and PrimPol (100 nM) was similar on undamaged DNA template. The percentage of dNMPs incorporated opposite the 1,2-GG CisPt CL is shown on a diagram.

To investigate whether PrimPol is capable of incorporating dNTPs opposite the 1,2-GG CisPt CL, we performed primer extension reactions in the presence of Mg^2+^ or Mn^2+^ ions. Two primer–template substrates were used: one with the cross-link immediately after the primer end (+ 1–2) and the other with the cross-link two nucleotides downstream (+ 3–4), allowing the polymerase to start with incorporation opposite undamaged DNA (Supplementary Figure 1 and Fig. 1A–D). In Mg^2+^-reactions, PrimPol was completely blocked at the base prior to the lesion in the + 3–4 (Fig. 1A, lanes 5–8) and + 1–2 positions downstream of the primer (Fig. 1B, lanes 5–8). Mn^2+^ ions significantly stimulated the DNA polymerase activity of PrimPol (Fig. 1A, lanes 9–12 and Fig. 1B, lanes 9–12) and allowed partial bypass of the 1,2-GG CisPt CL by PrimPol (Fig. 1A, lanes 13–16 and Fig. 1B, lanes 13–16). Noteworthy, PrimPol efficiently incorporated dNMPs opposite both guanines of the 1,2-GG CisPt CL placed at the + 1–2 and + 3–4 template positions but the extension beyond the DNA adducts was inhibited. The catalytically deficient PrimPol variant carrying the D114A substitution did not show the TLS activity opposite the lesion suggesting that this activity was intrinsic to PrimPol and not a result of contamination (Supplementary Figure 2).

We also compared the TLS activity of PrimPol with Pol β and Pol η. We used different concentrations of DNA polymerases in order to obtain similar levels of the DNA polymerase activity on undamaged DNA (Fig. 1D, lanes 4–6, 16–18 and 22–24). In reactions supporting equal levels of catalytic activity, the dNMPs incorporation opposite the 1,2-GG CisPt CL by PrimPol (Fig. 1D, lanes 1–3, 7–9) was slightly more efficient compared to Pol β (Fig. 1D, lanes 13–18) but was less efficient compared to Pol η (Fig. 1D, lanes 19–24) (Mn^2+^-containing buffer was used for PrimPol and the buffer with Mg^2+^was used for Pol β and Pol η).

We also tested the incorporation of individual dNMPs on a DNA substrate with the 1,2-GG CisPt CL (Fig. 1C). PrimPol preferentially incorporated two complementary dCMPs opposite the 3′-G (+ 1G) and 5′-G (+ 2G) of the 1,2-GG CisPt CL (Fig. 1C, lane 10), and also incorporated one dTMP opposite the 3′-G (Fig. 1C, lane 9). The ratio of dCMP/dTMP incorporation was 5.6 and 2.3 for undamaged DNA and 1,2-GG CisPt CL, respectively. It is likely that PrimPol incorporates pyrimidine nucleotides directly opposite the 1,2-GG CisPt CL avoiding lesion skipping because the lesion is not flanked by adenines and guanines in DNA template. PrimPol also incorporated two dGMP opposite the 1,2-GG CisPt CL with low efficiency (Fig. 1D, lanes 3 and 8 shows) which could be a result of skipping two T and incorporation opposite two C in the downstream template sequence 3'-TTCC.

PrimPol extended the 3′-primer ends with two C or one C paired with the 1,2-GG CisPt CL by incorporating dAMP, dTMP or dCMP and one T paired with the 3′-G of the lesion by incorporating dCMP or dGMP (Supplementary Figure 3A, B, C). The stretches of C and A on DNA template with the lesion were observed. It is likely that multiple dCMP and dAMP insertions resulting in primer expansion are mediated by primer dislocation and slippage and can be templated by the 1,2-GG CisPt CL and + 2–3 TT.

PrimPol was also able to incorporate ribonucleotides opposite the 1,2-GG CisPt CL with low efficiency (at high protein concentration) and preferentially incorporated complementary rCMP (Supplementary Figure 3D).

### Efficient 1,2-GG cisplatin CL bypass by PrimPol in cooperation with PolDIP2 and Pol ζ

**Figure 2:**
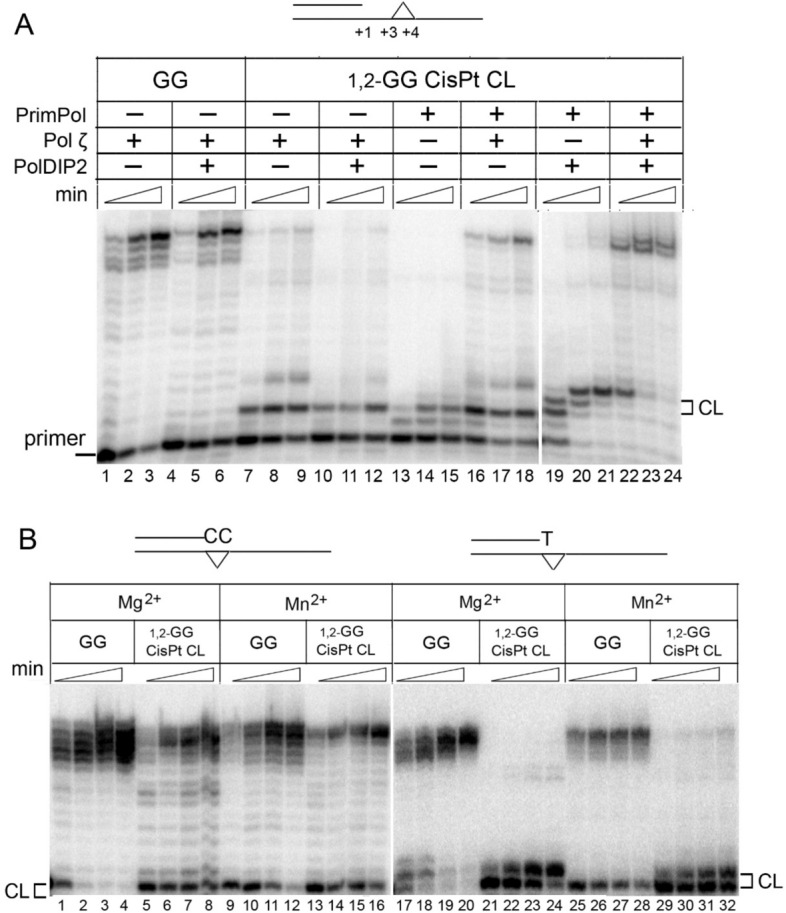
(**A**) The TLS on DNA substrate with the 1,2-GG CisPt CL by PrimPol in combination with Pol ζ. Reactions were carried out on a DNA substrate with the 1,2-GG CisPt CL at the + 3–4 position downstream of the primer in the presence or in the absence of PolDIP2. (**B**) Extension of correctly paired and mispaired primer termini by Pol ζ. Extension reactions were carried out on DNA substrates with matched primer termini (CC) paired with undamaged GG or the 1,2-GG CisPt CL and mismatched primer with the 3′-end T paired with the 3′-G of the 1,2-GG CisPt CL. Reactions were incubated for 2, 5, 10 and 20 min in the presence of 10 mM MgCl_2_ or 0.5 mM MnCl_2_.

Accessory protein PolDIP2 stimulates the DNA polymerase and strand displacement activities of PrimPol^16,38^. We tested whether PolDIP2 stimulates the TLS activity of PrimPol across the 1,2-GG CisPt CL (Fig. 2). The full-length nuclear isoform of PolDIP2 was used in experiments. PolDIP2 stimulated the DNA polymerase activity of PrimPol on undamaged DNA and has no contaminating polymerase activity (Supplementary Figure 4). PolDIP2 significantly simulated the incorporation of dNMPs opposite the 1,2-GG CisPt CL (Fig. 2, lanes 19–21) but did not improve the extension beyond the lesion.

DNA polymerases Pol ζ and, in some cases, Pol κ efficiently extend DNA primers following nucleotide incorporation at the sites of DNA damage (“extender” polymerases)^39–42^. We performed series of primer extension experiments on a DNA template with the 1,2-GG CisPt CL in the presence of PrimPol and combinations of PolDIP2, human Pol κ (Supplementary Figure 5) and yeast Pol ζ, which is highly similar to human Pol ζ (Fig. 2A). Pol κ and Pol ζ carried out efficient full-length DNA synthesis on the undamaged template (Fig. 2, lanes 1–3 and Supplementary Figure 5, lanes 1–3) and partially bypassed the 1,2-GG CisPt CL (Fig. 2, lanes 7–9 and Supplementary Figure 5, lanes 7–9). PolDIP2 slightly inhibited Pol κ (Supplementary Figure 5, lanes 10–12) and Pol ζ activity (Fig. 2A, lanes 10–12).

Next, we performed primer extension reactions with sequential addition of PrimPol (together with PolDIP2) and an extender polymerase: reactions containing PrimPol were initiated with dNTPs, and Pol κ or Pol ζ was added after 10 min. Pol ζ (Fig. 2A, lanes 16–18) efficiently extended from the PrimPol-generated primer paired with the 1,2-GG CisPt CL. Moreover, addition of Pol ζ to reactions containing PolDIP2 reduced a pause at the 3′-G of the 1,2-GG CisPt CL and significantly improved DNA lesion bypass (Fig. 2A, lanes 22–24). Therefore, the synergistic effect in the bypass of the 1,2-GG CisPt CL in vitro was observed between PrimPol and Pol ζ (in the presence of PolDIP2). Importantly, Pol ζ efficiently extended the correctly paired primer with the 3′-end CC opposite the lesion and primer with the 3′-end noncomplementary T paired with the undamaged G but paused on DNA substrate containing the mismatched primer with T paired with the 3′-G of 1,2-GG CisPt (Fig. 2B). Pol κ in combination with PolDIP2 moderately increased the efficiency of the 1,2-GG CisPt CL bypass (Supplementary Figure 5).

CPD is another well-studied example of a cross-link between two adjacent DNA bases. Some translesion DNA polymerases, e.g., Pol η, efficiently incorporate dNMPs opposite both the 1,2-GG CisPt CL^43^ and CPD^44^. In this work, we also analyzed the TLS activity of PrimPol on DNA substrate with CPD. Unlike Pol η, PrimPol alone could not incorporate dNMPs on a DNA substrate with CPD even in the presence of Mn^2+^ ions (Supplementary Figures 6A and 6B, lanes 13–15). PolDIP2 improved the TLS activity of PrimPol on a DNA substrate with CPD, and partial bypass of CPD was observed in the reactions with PolDIP2 (Supplementary Figures 6A and 6B, lanes 19–21). However, no synergetic effect between PrimPol and Pol ζ or Pol κ was observed either in combination with PolDIP2 or in the absence of PolDIP2 (Supplementary Figures 6A and 6B, lanes 16–18, 22–24). Moreover, the TLS by Pol ζ was inhibited in the presence of PrimPol and PolDIP2 suggesting that primer termini produced by PrimPol on DNA template with CPD are not a good substrate for elongation by Pol ζ.

### The effect of Arg47 and Arg76 mutations on the 1,2-GG CisPt CL bypass

In PrimPol, the active site residues Arg47 and Arg76 contact a phosphate group and the templating base, respectively^45^, and might play a role in the DNA damage bypass. Recently, we have demonstrated that substitutions of either residue attenuate the catalytic activities of PrimPol and formation of the stable PrimPol:DNA complex in the presence of nucleotide substrates^46^. Mutations of both residues also affected the dNMPs incorporation opposite 8-oxoG and undamaged DNA templates. Moreover, it was suggested that the Arg76 residue affects the accommodation of distorting DNA adducts (e.g. CPD) in the active site of PrimPol^45^.

**Figure 3:**
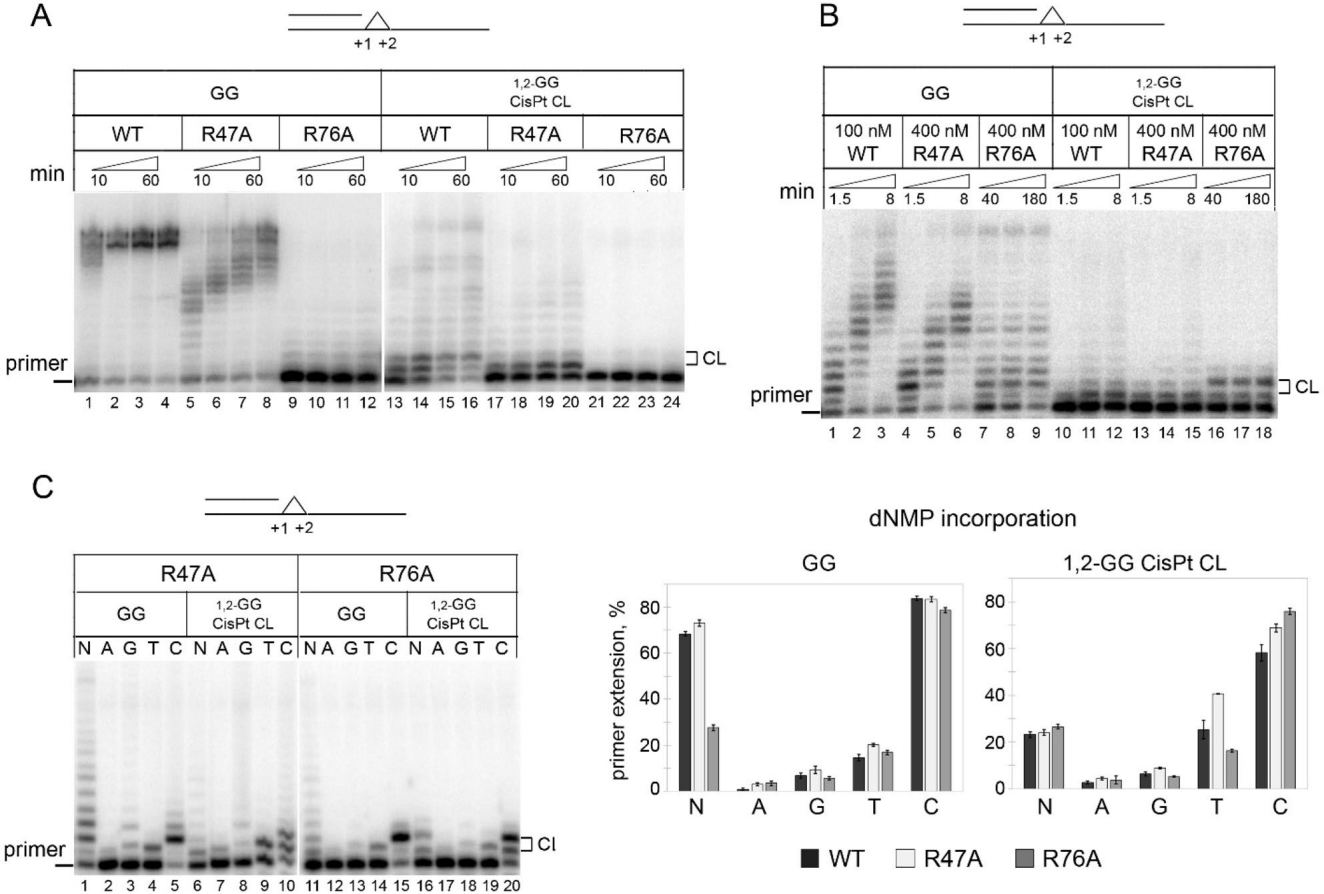
The TLS activity of the R47A and R76A PrimPol variants on DNA substrates with the 1,2-GG CisPt CL. (**A**) The DNA polymerase activity of PrimPol mutant variants on DNA substrates with the 1,2-GG CisPt CL or undamaged GG at the + 1–2 positions. Reactions were carried out in the presence of 200 nM wild-type PrimPol or 200 nM of mutant PrimPol variant for 10, 20, 40 or 60 min. (**B**) The adjusted DNA polymerase activity of PrimPol mutant variants on DNA substrates with the 1,2-GG CisPt CL or undamaged GG at the + 1–2 positions. Reactions were carried out in the presence of 100 nM wild-type PrimPol for 1.5, 4 or 8 min, 400 nM R47A variant for 1.5, 4 or 8 min and 400 nM R76A variant for 40, 80 and 180 min. (**C**) dNMPs incorporation opposite undamaged GG or 1,2-GG CisPt CL by the R47A and R76A PrimPol variants. Reactions were carried out in the presence of 200 nM PrimPol for 1 and 4 min for the wild-type PrimPol, 4 and 8 min for the R47A variant and 10 and 80 min for the R76A variant opposite GG and 1,2-GG CisPt CL, respectively.

In this work, we analyzed the role of the Arg47 and Arg76 in the TLS activity of PrimPol on DNA with the 1,2-GG CisPt CL. As expected, the R47A substitution decreased the DNA polymerase activity of PrimPol on undamaged and damaged DNA templates whereas the R76A substitution caused almost complete loss of catalytic activity (Fig. 3A). In reactions with equal PrimPol concentrations and time, the R47A substitution decreased the incorporation of dCMP opposite the 5′-G of the lesion compared to the wild-type PrimPol (Fig. 3A, lanes 17–20). We increased protein concentration and incubation time to facilitate nucleotide incorporation by the R76A PrimPol mutant to the activity level observed for the wild-type protein. Interestingly, the Arg76 mutation increased the length of DNA products on undamaged DNA (Fig. 3B, lanes 1, 4 and 7) and improved the incorporation of dCMP opposite the 5′-G of the 1,2-GG CisPt CL (Fig. 3B, lanes 16–18). The R76A substitution also decreased the incorporation of noncomplementary dTMP opposite the 3′-G of the lesion (Fig. 3C). The ratio of dCMP/dTMP incorporation was similar (4.6–4.8) for both undamaged G and the 1,2-GG CisPt CL. These data suggest that the active site residues Arg47 and Arg76 might be important for positioning of damaged nucleobases and facilitate (Arg47) or hinder (Arg76) DNA lesion bypass.

### DpCLs block PrimPol

**Figure 4:**
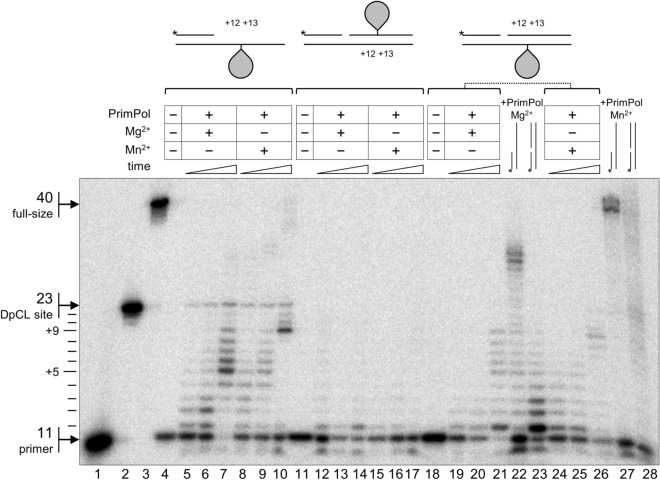
DNA polymerase and strand displacement activities of PrimPol on DNA substrate with a DpCL. Schemes of the DpCL-containing substrates are shown at the top panel. The CL site is located at the + 13 position from the 3ʹ-OH of the primer. *Lanes 1–3*, size markers corresponding to the primer (*lane 1*, 11 nt long), primer extended to the position immediately before the cross-link site (*lane 2*, 23 nt) and full-size product (*lane 3*, 40 nt). *Lane 4*, ss-DpCL without PrimPol, *lanes 5–7*, ss-DpCL with PrimPol and Mg^2+^, *lanes 8–10*, ss-DpCL with PrimPol and Mn^2+^. *Lane 11*, down-DpCL without PrimPol, *lanes 12–14*, down-DpCL with PrimPol and Mg^2+^, *lanes 15–17*, down-DpCL with PrimPol and Mn^2+^. *Lane 18*, temp-DpCL without PrimPol, *lanes 19–21*, temp-DpCL with PrimPol and Mg^2+^, *lanes 24–26*, temp-DpCL with PrimPol and Mn^2+^. In all cases, PrimPol extension time points were 2, 5, and 30 min. In *lanes 22* and *27*, the reaction was carried out with an undamaged primer–template substrate in the presence of Mg^2+^ (*lane 22*) or Mn^2+^ (*lane 27*), and in *lanes 23 and 28*, with an undamaged primer–downstream strand–template substrate in the presence of Mg^2+^ (*lane 23*) or Mn^2+^ (*lane 28*) for 30 min.

Despite PrimPol’s ability to bypass or “skip” a variety of blocking DNA lesions (e.g. εA, CPD, T–T (6–4) photoproducts)^2,5,10,12^, full-size DpCLs may present an impassible barrier, as was shown for a variety of DNA polymerases from families A, B, X, and Y^33^. However, the TLS` activity of PrimPol on DNA with extremely large obstacles such as DpCLs in the template strand is yet to be investigated. Moreover, PrimPol possesses weak strand displacement activity^38^, but no data is availible on the situation when PrimPol displaces downtream strand carrying a DpCL, which is also blocking for other DNA polymerases notwithstanding the undamaged template^33^. Therefore, we investigated the ability of PrimPol to perform synthesis on the substrates containing a DpCL in the template strand of single-stranded DNA (ss-DpCL), in the template strand of double-stranded DNA (temp-DpCL) or in the displaced strand of double-stranded DNA (down-DpCL) (Fig. 4). To create a model DpCL with a defined structure at a pre-determined position, we used the ability of the bifunctional DNA glycosylase Fpg to form a Schiff base reaction intermediate that can be reduced by NaBH_4_ to produce a stable covalent DNA-Fpg conjugate. The CL site was created at the position + 13 from the primer end.

PrimPol was almost fully blocked by an ss-DpCL, and the DNA synthesis was terminated at the position + 12, immediately before the CL site, in the presence of either Mg^2+^ or Mn^2+^ ions (Fig. 4, lanes 5–10). Additionally, pauses at the positions + 5 in the Mg^2+^ reactions (Fig. 4, lanes 5–7) and + 9 in the Mn^2+^ reactions (Fig. 4, lanes 5–10) were observed. These primer elongation termination sites located, respectively, 8 and 4 nt upstream of the ss-DpCL can be caused by a clash between the surfaces of PrimPol and Fpg. Interestingly, weak but reproducible bands corresponding to the species longer than 23 nt (primer extended to the position immediately before CL) were observed in the presence of Mn^2+^ ions (Fig. 4, lanes 8–10), which might be an evidence of partial ss-DpCL bypass by PrimPol, perhaps by template scrunching.

Remarkably, the ability of PrimPol to elongate the primer with downstream strand displacement strongly depended on the position of the DpCL obstacle ahead. On double-stranded DNA with a DpCL in the template strand, PrimPol possessed weak strand displacement activity and stopped mainly at the position + 9 (4 nt upstream of the CL site; Fig. 4, lanes 19–21 and 24–26). This was comparable with the ability of PrimPol to perform synthesis with strand displacement in the absence of a DpCL (Fig. 4, lane 23). Incorporation of a DpCL in the downstream strand of double-stranded DNA dramatically reduced the efficiency of DNA synthesis with DNA strand displacement by PrimPol in the presence of either Mg^2+^ or Mn^2+^ ions (Fig. 4, lanes 12–17). No elongation beyond the CL site was observed.

## Discussion

### The TLS activity of PrimPol opposite the 1,2-GG cisplatin CL

Human PrimPol is very special among DNA polymerases. It possesses the DNA primase and DNA polymerase activities^1,2^, anneals DNA strands based on microhomology^12^ and bypasses DNA lesions by incorporating nucleotides opposite damage or by skipping the lesion^1,2,5,10–12^. Avian and human PrimPol-deficient cells demonstrate elevated sensitivity to cisplatin treatment that indicates the role of PrimPol in the tolerance of cisplatin-induced DNA damage^3,4,9^. It was suggested that PrimPol re-initiates stalled replication forks on the leading DNA strand at the sites of blocking DNA lesions such as photoproducts or cisplatin adducts using the DNA primase activity^4,5,9^. However, the situation is possible when during de novo DNA synthesis PrimPol encouners a cisplatin adduct. In this work, we have analyzed the properties of PrimPol on DNA bearing the most common intrastrand cisplatin adduct, 1,2-GG CisPt CL.

The 1,2-GG CisPt CL locally disrupts DNA structure, unwinding the helix and bending the duplex toward the major groove^47^. It represents a strong block to eukaryotic replicative DNA polymerases Pol α, Pol δ, and Pol ε^19^ and repair and translesion DNA polymerases Pol ι and Pol λ^48,49^. Pol γ is significantly blocked opposite the 1,2-GG CisPt CL and frequently misincorporates dAMP opposite the 3′G^50,51^. Pol β preferably incorporates complementary dCMP opposite the 3′-G and 5′-G with low efficiency but also misincorporates dTMP and dAMP opposite 5′-dG of the 1,2-GG CisPt CL^51–53^. Pol η is the most efficient and accurate polymerase opposite the 1,2-GG CisPt CL: Pol η preferentially incorporates dCMPs opposite the 3′-G and 5′-G but extension beyond the lesion requires an extender polymerases Pol ζ or Pol κ^41,51^.

In this work, we demonstrate that PrimPol is able to incorporate nucleotides opposite the 5′-G and 3′-G of the 1,2-GG CisPt CL in the presence of Mn^2+^ ions, and that its activity is stimulated by the accessory protein PolDIP2. Recently, it was shown that PolDIP2 enhances PrimPol binding to DNA^16^, stimulates the processivity of DNA synthesis^16^ and strand displacement activity^38^ as well as increases efficiency and fidelity of 8-oxoG bypass by PrimPol^16^. Our data also support the role of PolDIP2 in the stimulation of the TLS activity of PrimPol. Moreover, PrimPol efficiently synthesized through the 1,2-GG CisPt CL with an assistance of an extender polymerase Pol ζ. Complete bypass of cisplatin lesion in vitro required PrimPol, PolDIP2 and Pol ζ. In this work, we used yeast four-subunit Pol ζ. Human Pol ζ efficiently extends primer termini pairied with the 1,2-GG CisPt CL^41^ and its role in resistance to cisplatin and platinum based chemotherapy is well established^54,55^. Yeast Pol ζ is shown to be an efficient extender of mismatches created by a variety of DNA lesions including DNA crosslinks^56–58^. Thus, we argue that the results obtained with yeast Pol ζ can be relevant for human Pol ζ. A role of Pol κ in tolerance to cisplatin was also suggested^59^. Pol κ inefficiently incorporates nucleotides opposite the 1,2-GG Cis-Pt CL but efficiently and accurateley extends DNA primers located at the 3′- or 5′-G of the lesion^60^. Here, we also showed that Pol κ can extend from primer ends generated by PrimPol across from 1,2-GG CL but performs this less efficiently than Pol ζ.

Altogether, our data show that PrimPol and Pol ζ carry out fairly accurate TLS acting in concert to bypass the 1,2-GG CisPt CL in vitro. However, the role of the TLS activity of PrimPol opposite cisplatin adducts in vivo is not clear yet. On one hand, chicken *PRIMPOL*^*−/−*^ DT40 cells are hypersensitive to cisplatin but primase-deficient PrimPol does not suppress the hypersensitivity of *PRIMPOL*^*−/−*^ cells sugesting that its primase, rather than TLS activity, is pivotal for DNA damage tolerance^4^. In human BRCA1-deficent cells, PrimPol promotes repriming and accumulation of internal ssDNA gaps behind forks after cisplatin treatment^9^. Moreover, PrimPol and Pol ζ contribute to DNA damage tolerance independently of each other in avian cells^4^. On the other hand, PrimPol could contribute to the tolerance of some sub-pool of cisplatin adducts. In this respect, it is interesting that higher amounts of cisplatin–DNA adducts and their poor removal were reported for mitochondrial DNA as compared to nuclear DNA^61^. Since Pol γ is not efficient in replicating opposite the 1,2-GG CisPt CL^50^, but both PrimPol and Pol ζ are present in mammalian mitochondria^62^, it is conceivable that the observed TLS activity of PrimPol may play a role in cisplatin tolerance in mitochondria, in addition to the repriming downstream of cisplatin adducts. Future research is needed to explore possible functional interactions between PrimPol and Pol ζ and the role of PrimPol in TLS across cisplatin adducts in mitochondria.

Like the 1,2-GG CisPt CL, the UV-induced cyclobutane T-T dimer represents an example of a distorting lesion with crosslinked adjacent nucleobases. The efficient TLS activity of PrimPol on the 1,2-GG CisPt CL in the presence of Mn^2+^ ions was unexpected because the active site of PrimPol does not have enough space for accomodation of CPD and (6–4) T-T photoproducts^45^. The PrimPol active site is relatively constrained in a CPD accommodation model: the 5ʹ-T of the dimer clashes with Gly74 and Arg76 and is forced out of the active site cleft^45^. We analyzed the efficiency of the TLS by PrimPol on a DNA template with CPD in control experiments. Indeed, unlike Pol η, which efficiently incorporates dNMPs opposite the 1,2-GG CisPt CL and CPD adducts, PrimPol lacked the TLS activity on DNA template with a CPD. In particular, PrimPol failed to incorporate nucleotides opposite CPD and demonstrated poor CPD bypass in the presence of PolDIP2. It is likely that the TLS activity of PrimPol on DNA substrate with CPD observed in some studies is mediated by lesion skipping and depends on the sequence context of the lesion^2,5,12^.

In this work, PrimPol preferentially incorporated two complementary dCMPs opposite guanines of the 1,2-GG CisPt CL or inserted one dTMP opposite the 3′-G of the adduct. We suggest that this TLS activity is not mediated by a lesion skipping because ssDNA template lacks microhomology with the 3′-C and 3′-T primer nucleotides. The mechanism of PrimPol inhibition opposite the 1,2-GG CisPt CL in the presence of Mg^2+^ ions can be similar with CPD and (6–4) T-T photoproduct^5^ and can involve overlapping of the 5ʹ-base of the crosslink with the side chain of Gly74, Gln75 or Arg76. Indeed, mutation of the Arg76 residue improved the incorporation of complementary dCMP opposite the 5′-G on the lesion at the expense of T misincorporation. Structural studies are requried to investigate the mechanism of the 1,2-GG CisPt CL bypass by PrimPol in more detail.

### The DNA polymerase and strand displacement activities of PrimPol on DNA with a DpCL

DNA polymerases usually cannot traverse cross-linked protein molecules^33,35,63,64^. However, the behavior of PrimPol or any other AEP superfamily enzyme upon an encounter with a DpCL has never been addressed before. Given the ability of PrimPol to skip strongly blocking base lesions by template scrunching, it was interesting whether the same mechanism could be operating on very bulky DpCLs.

PrimPol demonstrated poor ability to synthesize past the CL site. The best substrate was a primer–template structure with a DpCL in the single-stranded template, in which PrimPol was able to extend the primer until the position immediately before the CL site. Compared with other polymerases, this ability puts PrimPol into one group with B family DNA polymerases from phages (T4 and RB69 polymerases), which can also efficiently utilize a single-stranded template to the last normal nucleotide before a DpCL^33^. However, pause points were observed a few nucleotides before the CL site. The main factor in stopping the DNA synthesis through DpCL seems to be a clash between the surfaces of an elongating polymerase and the protein obstacle, followed by the protein globule deformation up to the point of incompatibility with substrate binding or catalysis (the “kiss-and-push” model^33,65^). The structure of PrimPol ternary complex with a primer–template and an incoming dNTP^45^ suggests that single-stranded template is kinked by ~ 90° when exiting the enzyme’s active site. Such an arrangement would presumably minimize collisions of the polymerase with the protein part of a DpCL, allowing these two molecules to approach each other closely, while the pause points may correspond to the sites where a steric clash still occurs.

PrimPol demonstrated a moderate ability to displace an undamaged downstream strand but showed striking asymmetry when a DpCL was present in double-stranded DNA. While a DpCL in the template strand decreased the overall efficiency of primer elongation and did not allow the polymerase to reach the CL site, a DpCL in the downstream strand almost completely abrogated strand displacement. This cannot be explained by differences in the CL footprint: judging from the structure of *E. coli* Fpg cross-linked to DNA^66^, the primer end is 10 nt away from the nearest Fpg surface if the cross-link is in the displaced strand and 8 nt away if it is in the template strand. Although other DNA polymerases cannot pass the Fpg DpCL in the displaced strand, they usually elongate the primer in such substrate better than in the double-stranded template DpCL substrate^33^. This difference may suggest that PrimPol interacts with the downstream DNA in a unique way compared with other DNA polymerases, perhaps extending much farther in the 3′-direction; a structure of PrimPol with a gapped substrate would be required to unravel this conundrum.

In the presence of Mn^2+^, elongating PrimPol was able to come closer to the DpCL in a single- or double-stranded template compared with Mg^2+^-dependent reactions. Although Mn^2+^ is regarded as a divalent cation that lowers the fidelity of DNA polymerases, it does so by increasing both the affinity of polymerases for dNTPs and the efficiency of the catalytic step^11,67^. Therefore, the increased overall efficiency of catalysis in the presence of Mn^2+^ may promote the advance of PrimPol despite unfavorable steric interactions with the DpCL. Some minor fraction of the polymerase might get hold on the template at the other side of the cross-linked protein molecule and insert a few dNMPs, essentially skipping the obstacle. It remains to be seen whether PrimPol can reprime DNA synthesis beyond a DpCL. Alternatively, this product may reflect a switch of the polymerase into the template-independent mode, which was demonstrated for PrimPol in the presence of Mn^2+^^68^.

## Methods

### Protein purification

HIS-SUMO-tagged human PrimPol was purified from *E. coli* Rosetta 2 strain. An overnight culture was grown at 30 °C and used to inoculate 4 L of LB medium. The culture was grown at 30 °C to OD_600_ = 0.4, then the temperature was reduced to 16 °C and expression was induced with 0.5 mM IPTG for 18 h. Cells were harvested by centrifugation at 9000 rpm for 5 min at 4 °C. Pellets were resuspended in lysis buffer A (20 mM Tris–HCl pH 7.9, 2% glycerol, 100 mM NaCl, 10 mM K_(2)_H_(2)_PO_4_, 4 mM β-mercaptoethanol, 10 µM pepstatin, 10 µM leupeptin, 2.5 mM benzamidine, 0.5 mM PMSF) and pulse-sonicated on a Cole Parmer ultrasonic homogenizer for 20 min (30% cycle, power 5–6). The cell lysates were clarified by centrifugation at 20,000 rpm for 30 min at 4 °C and incubated with Ni-Sepharose beads (GE Healthcare) for 4 h at 4 °C. The beads were loaded on an empty column and washed with buffer A, followed by washing with buffer B (20 mM Tris–HCl pH 7.9, 2% glycerol, 100 mM NaCl, 10 mM K_(2)_H_(2)_PO_4_, 4 mM β-mercaptoethanol, 0,01% NP-40), buffer C (20 mM Tris–HCl pH 7.9, 2% glycerol, 100 mM NaCl, 10 mM K_(2)_H_(2)_PO_4_, 4 mM β-mercaptoethanol, 1 mM ATP, 1 mM MgCl_2_) and buffer D (20 mM Tris–HCl pH 7.9, 2% glycerol, 400 mM NaCl, 40 mM K_(2)_H_(2)_PO_4_, 4 mM β-mercaptoethanol). After the final wash with buffer A, the protein was eluted with buffer E (20 mM Tris–HCL pH 7.9, 2% glycerol, 100 mM NaCl, 4 mM β-ME and 100 mM imidazole). The fractions containing PrimPol were combined and digested overnight at 4 °C with Ulp protease to remove the SUMO tag. The eluate was dialyzed against buffer F1 (20 mM Tris–HCL pH 7.9, 2% glycerol, 100 mM NaCl, 4 mM β-mercaptoethanol) and loaded onto three 1-ml columns (SP HP, His-trap HP, and heparin HP, 1 ml each, GE healthcare) connected in a series. After washing the columns with buffer F1, SP and His columns were disconnected, and a linear salt gradient from buffer F1 to buffer F2 (20 mM Tris–HCL pH 7.9, 2% glycerol, 1000 mM NaCl, 4 mM β-ME) was applied to elute PrimPol from the heparin column. PrimPol was eluted at 200 mM NaCl. Protein samples were aliquoted, frozen in liquid nitrogen and stored at − 80 °C. PrimPol D114A mutant variant was obtained by site-directed mutagenesis and purified as the wild-type enzyme. PrimPol R47A and R76A mutant variants were purified as described^46^.

The full-length human PolDIP2 was purified from *E. coli* as described^38^. Four-subunit yeast Pol ζ was purified from 20 L of *Saccharomyces cerevisiae* culture as described^69^. To create the yeast expression vector encoding for human Pol η fused with the N-terminal GST-tag, the chemically synthesized and optimized to yeast codon usage *POLH* gene was cloned in the pRS424-GAL-GST-TRP plasmid under the chimeric GAL1-GAL10 promoter^70^. Pol η was purified from 10 L of *S. cerevisiae* culture using the Pol ζ protocol. Human Pol β was purified from 3 L of *E. coli* as described^71^. Human Pol κ was kindly provided by Dr. Leonid Gening (IMG, Moscow). *E. coli* formamidopyrimidine-DNA glycosylase (Fpg) was overexpressed and purified as described^66^. PrimPol, PolDIP2, Pol η and Pol ζ preparations are shown in Supplementary Figure 7.

### DNA oligonucleotide substrates

Unmodified DNA oligonucleotides were purchased from Syntol (Moscow, Russia). Oligonucleotides containing 8-oxoG were synthesized by the ICBFM Laboratory of Medicinal Chemistry (Novosibirsk, Russia). To obtain the substrates for DNA polymerase reactions, the primers were 5′-labeled with [γ-^32^P]-ATP by T4 polynucleotide kinase and annealed to the corresponding unlabeled template oligonucleotides. The sequences of the oligonucleotides used in this study are shown in Table 1 and the positions of cross-links are indicated.

**Table 1:** Oligonucleotides used in the study.

Template-CL	5ʹ-CCTCCTTCTCCTTGGTCATCTATCCCTTCT-3ʹ GG or 1,2-GG CisPt CL
Primer CL-13	5ʹ-AGAAGGGATAGAT-3ʹ
Primer CL-15	5ʹ-AGAAGGGATAGATGA-3ʹ
Primer CL-16 T	5ʹ-AGAAGGGATAGATGAT-3ʹ
Primer CL-16C	5ʹ-AGAAGGGATAGATGAC-3ʹ
Primer CL-17CC	5ʹ-AGAAGGGATAGATGACC-3ʹ
Primer DpCL-11	5ʹ-CGAGACCGTCG-3ʹ
Downstream_primer_oG DpCL-28	5ʹ-GAGGAAAGAAGXGAAGGAATTCCAGAGC-3ʹ X = 8-oxoG
Downstream_primer DpCL-28	5ʹ-GAGGAAAGAAGCGAAGGAATTCCAGAGC-3ʹ
Template_oG DpCL-40	5ʹ-GCTCTGGAATTCCTTCXCTTCTTTCCTCTCGACGGTCTCG-3ʹ X = 8-oxoG
Template DpCL-40	5ʹ-GCTCTGGAATTCCTTCCCTTCTTTCCTCTCGACGGTCTCG-3ʹ
Marker DpCL-23	5ʹ-CGAGACCGTCGCGAGGAAAGAAG-3ʹ
Marker DpCL-40	5ʹ-CGAGACCGTCGCGAGGAAAGAAGCGAAGGAATTCCAGAGC-3ʹ
Template-CPD	5ʹ-GGGCAGCTCAAGTAACTTGGCCTGGTCATT-3ʹ TT or *cis-syn* CPD
Primer CPD-11	5ʹ-AATGACCAGGC-3ʹ

### Preparation of the 1,2-GG cisplatin CL

Cisplatinated oligonucleotide was prepared according to^72^ with modifications. To replace chloride ligands by H_2_O (cisplatin activation), cisplatin (4.5 mg, 15 μmol, 1.0 mol eq, Sigma-Aldrich. St. Louis, MO) was dissolved in 980 μL H_2_O, mixed with 20 μl AgNO_3_ solution (1.5 M, 30 μmol, 2.0 mol eq) and incubated in the dark for 24 h at 37 °C and vigorous shaking (1000 rpm). The AgCl precipitate was centrifuged (13,000 rpm, 5 min) and the supernatant containing 15 mM activated cisplatin was collected. 100 μl of 1 mM single stranded DNA with a unique GG sequence (100 nmol, 1 mol eq) were mixed with 20 μl activated cisplatin solution (300 nmol, 3 mol eq) and 880 μl 113.6 mM NaClO_4_ pH 5.2 (final concentration 100 mM). The reaction mixture was incubated in the dark for 5 h at 37 °C.

Oligonucleotide containing a cisplatin adduct was purified by anion exchange FPLC as described previously^73^. Briefly, the DNA sample was diluted tenfold with buffer A (0.1 M NaCl, 10 mM Tris–HCl, pH 7.4) and loaded onto a Mono Q 5/50 GL column (GE healthcare). DNA reaction products were eluted by three-step gradient with buffer B (1 M NaCl, 10 mM Tris–HCl pH 7.4): 10–37% buffer B in 5 column volumes, 37–47% buffer B in 40 column volumes, and 47–100% buffer B in 1 column volume at a flow rate of 0.5 mL/min. The cisplatin adduct was eluted at 0.4 M NaCl in the first peak. The purity of platinated DNA was confirmed by 21%/8 M urea PAGE (Supplementary Figure 1).

### Preparation of DNA–protein cross-links

Model DpCLs were prepared as described in^33^. The reaction mixture contained 50 mM sodium phosphate (pH 6.8), 1 mM EDTA, 1 mM DTT and 30 pmol of one of the 8-oxoG-containing oligonucleotide substrates: single-stranded Template_oG DpCL-40 or duplexes Downstream_primer DpCL-28//Template_oG DpCL-40 and Downstream_primer_oG DpCL-28//Template DpCL-40, containing 8-oxoG in the template strand or in the downstream primer respectively. A 20-fold molar excess of Fpg and 100 mM NaBH_4_ were added simultaneously. The reaction was allowed to proceed for 1 h at 37 °C and stopped by adding glucose to 400 mM and incubating for 30 min on ice. The DNA–Fpg DpCL was purified by 8% native PAGE; the band of interest was excised, eluted overnight with 0.1 × TE buffer, and the solution was passed through a 0.22-μm pore filter (Ultrafree CL, Merck Millipore, Burlington, MA) and concentrated in a Centricon-10 ultrafiltration unit (Merck Millipore). The efficiency of cross-linking and the purity of DpCLs after purification and concentration was confirmed by SDS-PAGE. To complete the assembly of DpCLs containing substrate, an equimolar amount of the ^32^P-labeled primer (Primer DpCL-11) was added and incubated for 30 min at room temperature. As controls for DNA polymerase reactions, we used undamaged constructs: primer-template (Primer DpCL-11//Template DpCL-40) or primer-downstream primer-template (Primer DpCL-11 ~ Downstream_primer DpCL-28//Template DpCL-40).

### Primer extension reactions

Primer extension reactions were carried out in 20 μl of the reaction buffer containing 30 mM HEPES (pH 7.0), 5% glycerol, 0.1 mg/ml bovine serum albumin, 10 mM MgCl_2_ or 0.5 mM MnCl_2_, 20 nM DNA substrate, 200 μM dNTPs and 200 nM PrimPol (or 100 – 600 nM PrimPol) and 300 nM PolDIP2, 5 nM Pol κ or 30 nM Pol ζ. In control experiments, 10 nM Pol η or Pol β were used in the reactions. Reactions were started by adding dNTPs and were incubated at 37 °C for specified time (2–30 min). The reactions were terminated by adding 20 μl of loading buffer containing 95% formamide, 10 mM EDTA and 0.1% bromophenol blue. DNA products were heated at 95ºC for 2 min and resolved on 21% polyacrylamide gels containing 8 M urea, followed by phosphorimaging on Typhoon 9400 (GE Healthcare). Experiments were repeated 2–4 times.

## Supplementary information

Supplementary Information.

## Data Availability

The data that support the findings of this study are included in the Supplementary Information file or are available from the corresponding author on request.
