# Supplementary material for: Translesion activity of PrimPol on DNA with cisplatin and DNA–protein cross-links
Source: Sci Rep. 2021 Sep 2;11:17588. doi: 10.1038/s41598-021-96692-y (PMC8413282; doi:10.1038/s41598-021-96692-y)

Translesion activity of PrimPol on DNA with cisplatin and DNA-protein cross-links

Elizaveta O. Boldinova[1], Anna V. Yudkina[3], Evgeniy S. Shilkin[1], Diana I. Gagarinskaya[1], Andrey G. Baranovskiy[2], Tahir H. Tahirov[2], Dmitry O. Zharkov[3,4], Alena V. Makarova[1#]

[1] *Institute of Molecular Genetics, National Research Center «Kurchatov Institute», Kurchatov sq. 2, 123182, Moscow, Russia*
[2]*Eppley Institute for Research in Cancer, Fred & Pamela Buffett Cancer Center, University of Nebraska Medical Center, Omaha, NE, United States*
[3] *Institute of Chemical Biology and Fundamental Medicine, Siberian Branch of the Russian Academy of Sciences, 8 Lavrentiev Avenue, Novosibirsk 630090, Russia*
[4] *Novosibirsk State University, 2 Pirogova St., Novosibirsk 630090, Russia*

# corresponding author, E-mail address: amakarova-img@yandex.ru

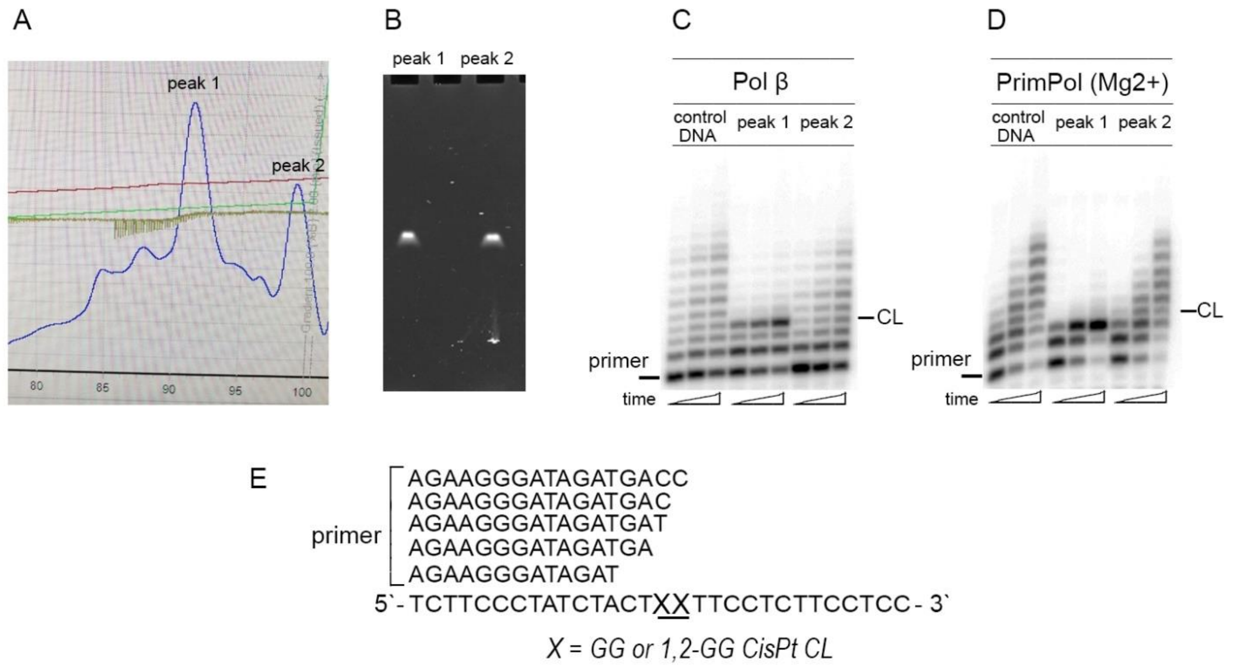

Supplementary figure 1. Purification and characterization of the DNA template with a 1,2-GG cisplatin CL. A. Purification of the DNA template (template-CL) with the 1,2-GG cisplatin CL by anion exchange FPLC. B. 21% 8M urea PAGE of platinated DNA oligonucleotides eluted at the peaks 1 and 2. C and D. The DNA polymerase activity of Pol β (C) and PrimPol (D) on control DNA substrate and DNA substrates containing platinated DNA oligonucleotides (template-CL) eluted at the peaks 1 and 2. Reactions were carried out for 2 – 15 min in the presence of 10 mM $MgCl_2$.

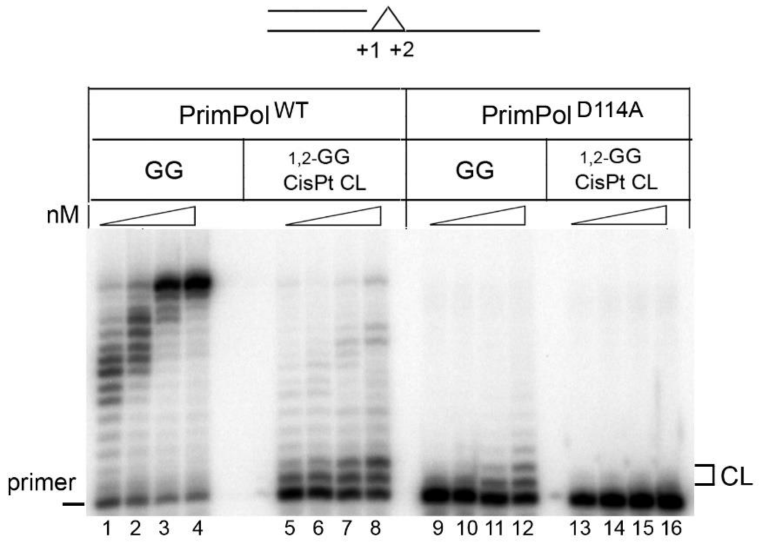

Supplementary figure 2. The TLS activity of the wild-type PrimPol and PrimPol with the D114A amino acid substitution on DNA substrates with the 1,2-GG CisPt CL at the +1–2 position downstream of the primer. Reactions were carried out in the presence of 100 – 600 nM PrimPol, 0.5 mM $MnCl_2$ for 10 min.

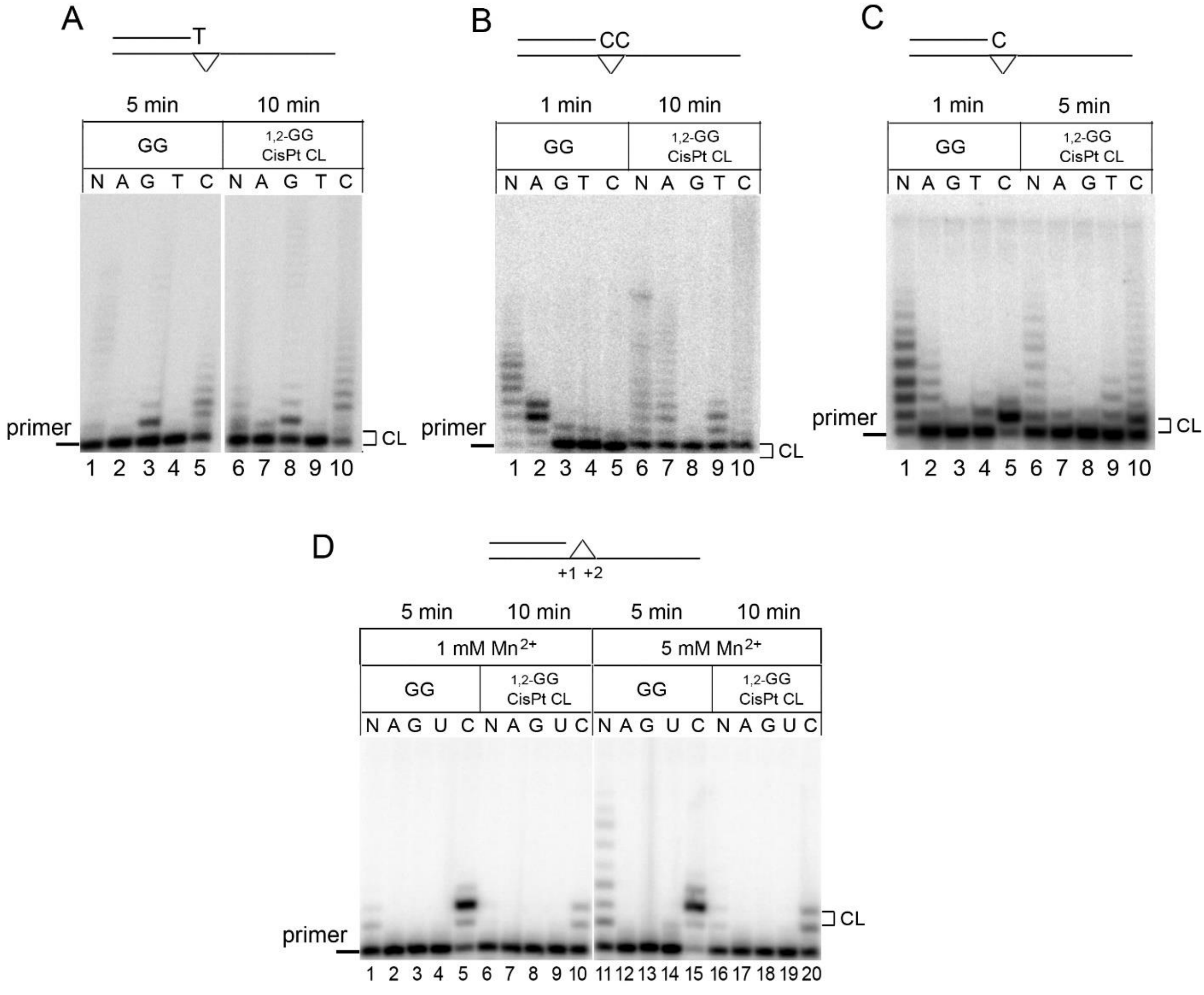

Supplementary figure 3. A. Extension of mispaired primer termini by PrimPol. Extension reaction was carried out on DNA substrates containing mismatched primer with the 3`-end T paired with the 3`-G of the 1,2-GG CisPt CL in the presence of 200 nM PrimPol and was incubated for 5 or 10 min. **B and C.**

Extension of correctly paired primer termini containing two C (B) or one C (C) by PrimPol. Extension reaction was carried out on DNA substrates containing primer with the 3`-end CC or C paired with the 1,2-GG CisPt CL in the presence of 200 nM PrimPol and was incubated for 1, 5 or 10 min **D.** Ribonucleotide incorporation opposite the 1,2-GG CisPt CL. Reactions were caried out in the presence of 400 nM PrimPol, 1 or 5 mM $MnCl_2$ and 200 µM of each rNMP, and incubated for 5–10 min.

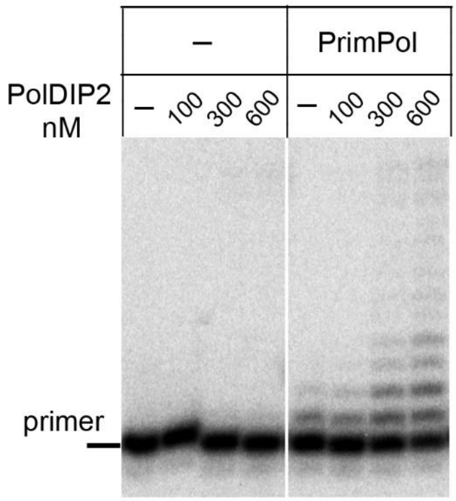

Supplementary figure 4. The DNA polymerase activity of PrimPol (100 nM) on undamaged DNA in the presence of PolDIP2. Reactions were carried out for 10 min in the presence of 10 mM $MgCl_2$.

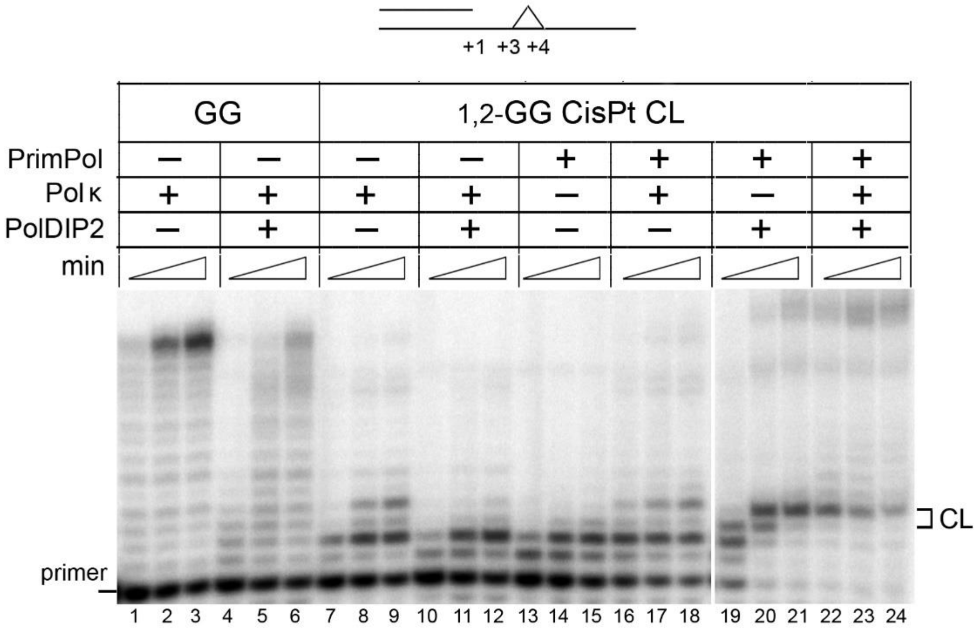

Supplementary figure 5. TLS on the DNA substrate with the 1,2-GG CisPt CL by PrimPol in combination with Pol κ. Reactions were carried out on DNA substrate with the 1,2-GG CisPt CL at the +3–4 position downstream of the primer in the presence or in the absence of PolDIP2.

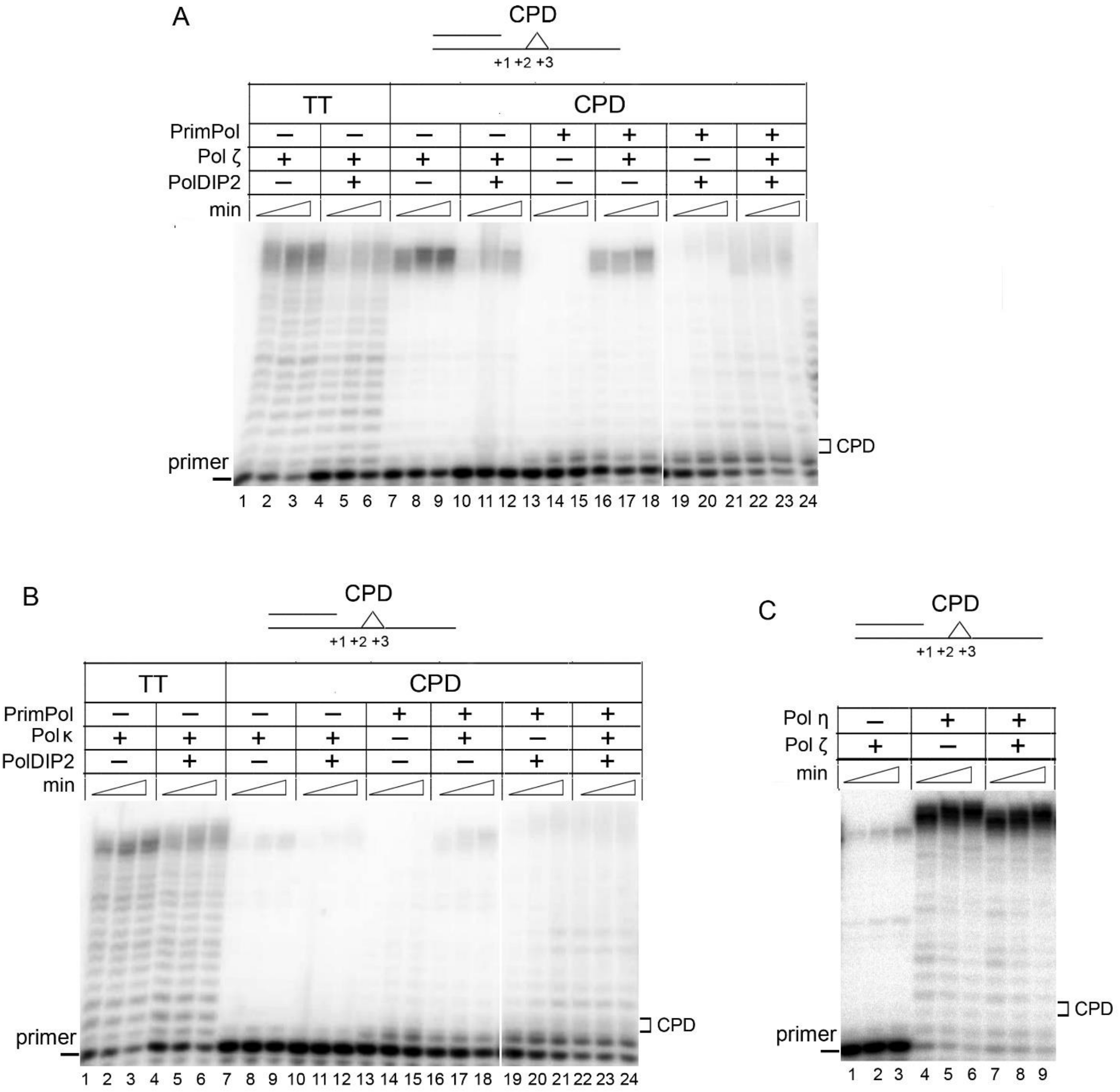

Supplementary figure 6. TLS on the DNA substrate with a CPD by PrimPol in combination with yeast Pol ζ (A) or Pol κ (B). Reactions were carried out on DNA substrate with a CPD at the +2–3 position downstream of the primer in the presence or in the absence of PolDIP2. C. Control TLS reactions on the DNA substrate with a CPD by Pol η.

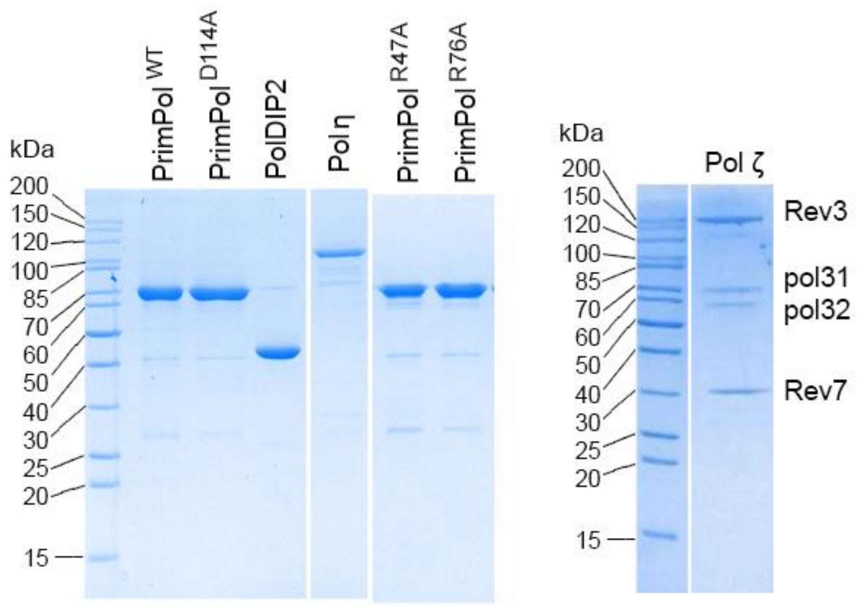

Supplementary figure 7. SDS-PAGE of the wild-type type and mutant PrimPol variants, PolDIP2, GST-Pol η and yeast Pol ζ. Coomassie staining.

Figure 1A

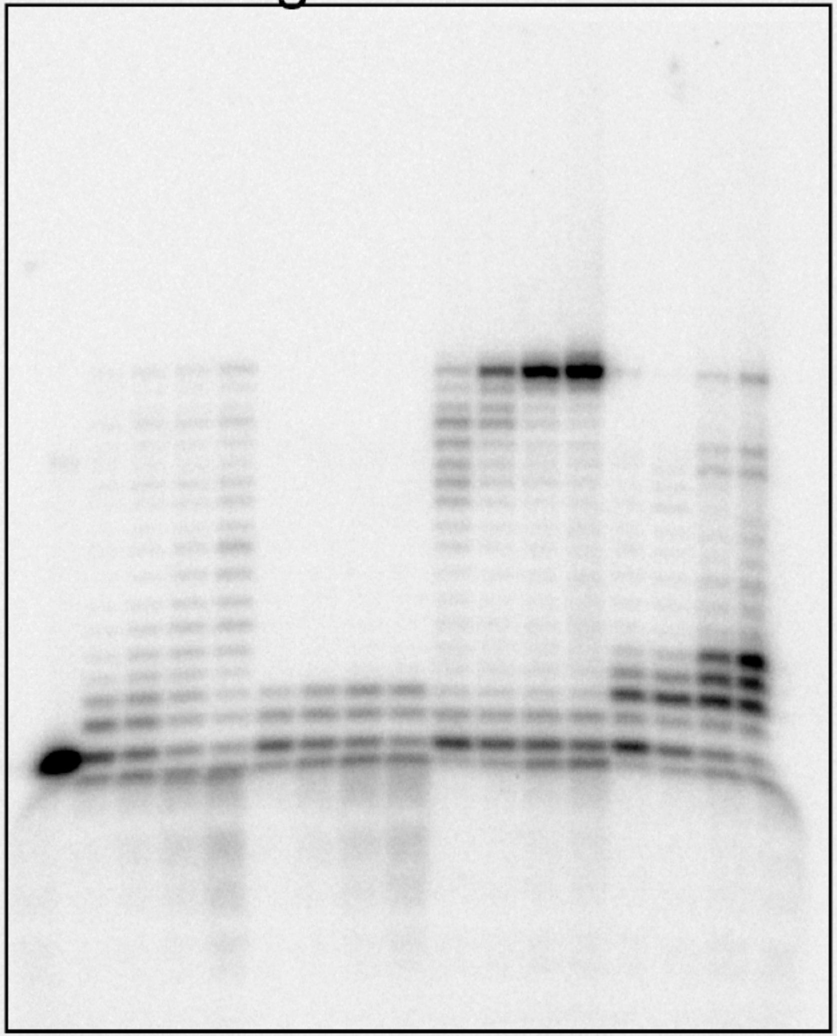

Figure 1B

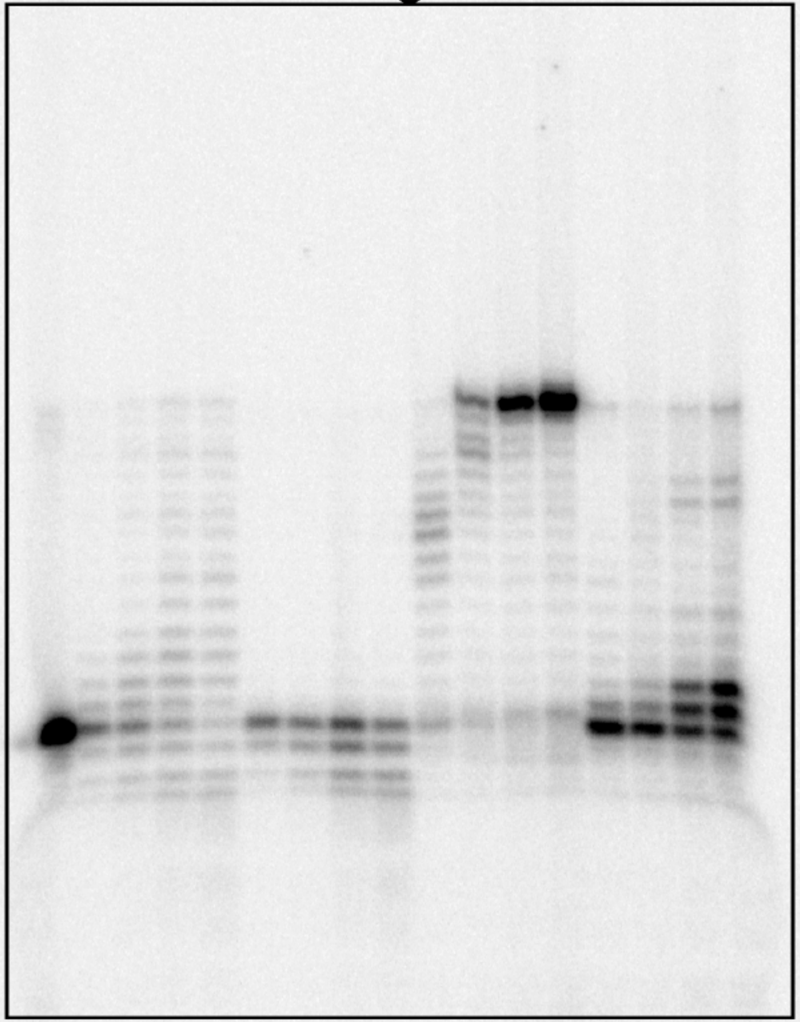

Figure 1C

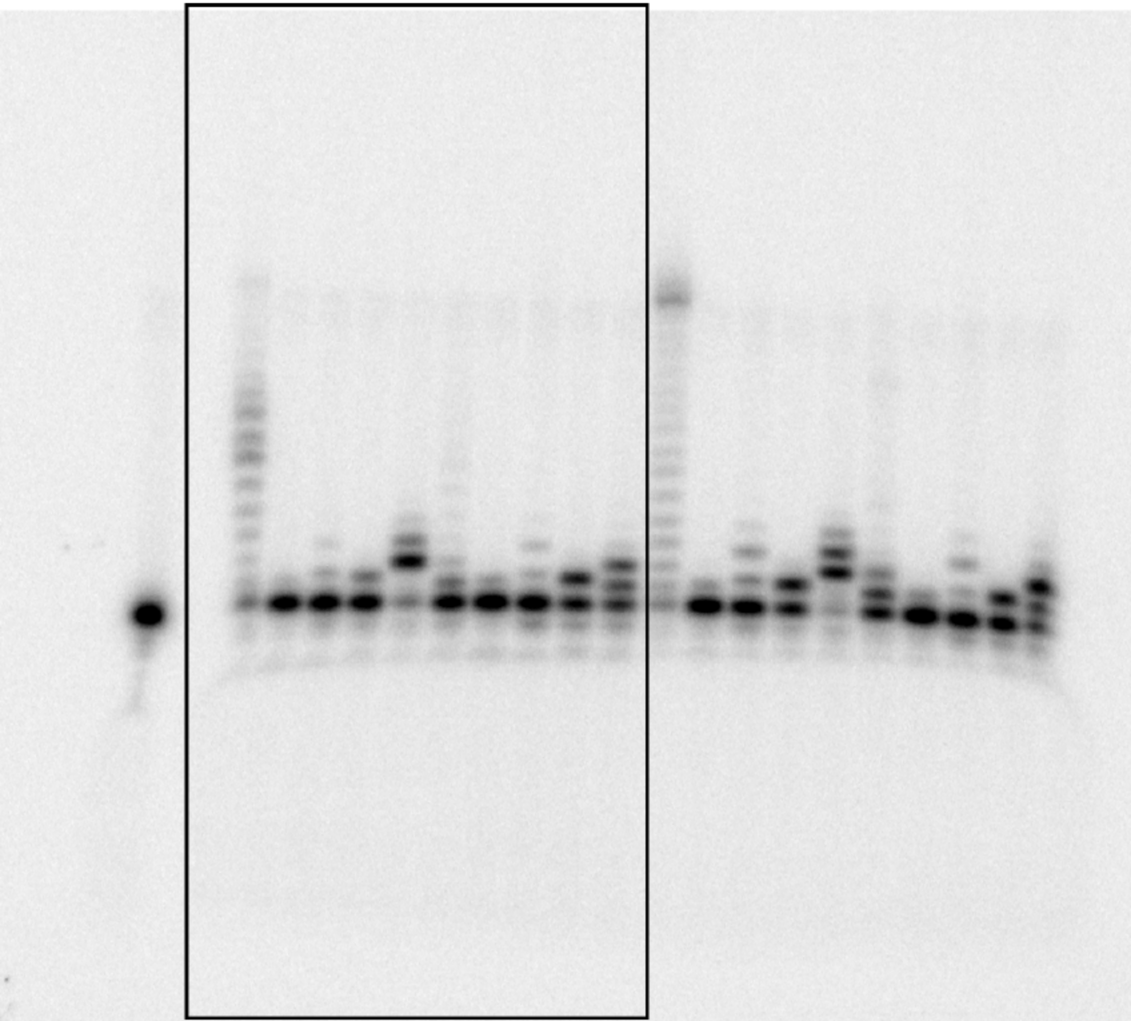

Figure 1D

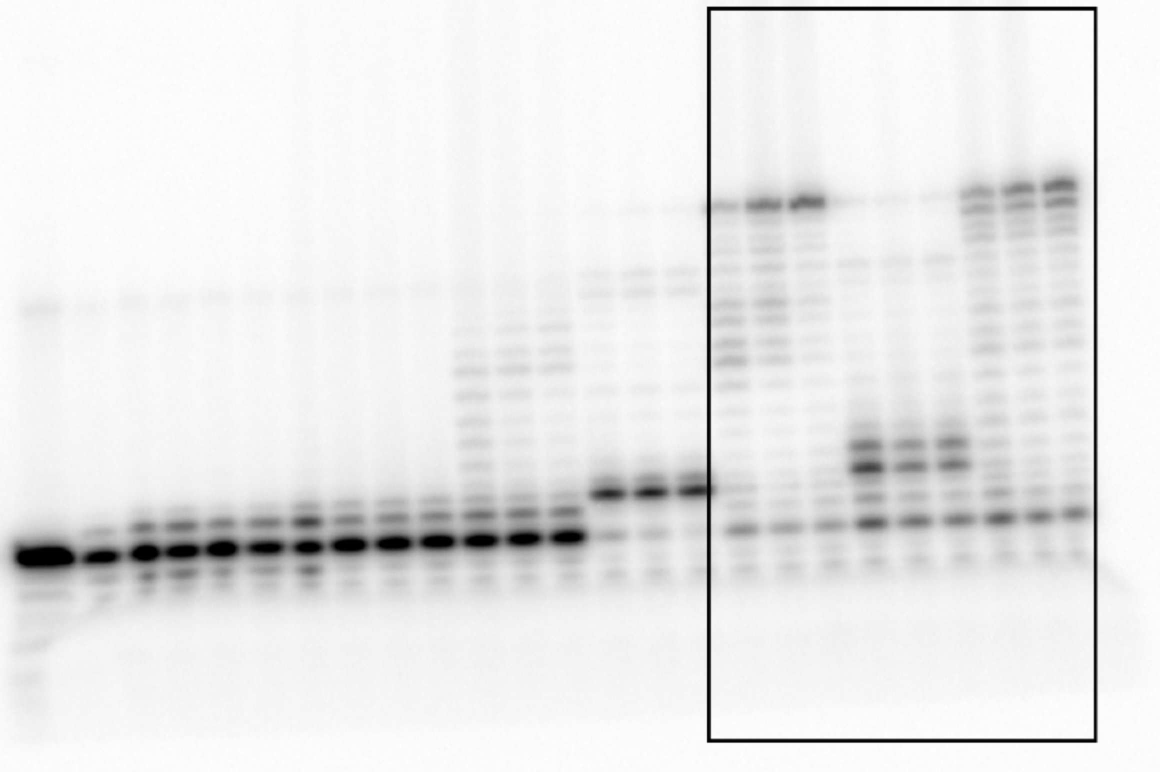

Figure 1D

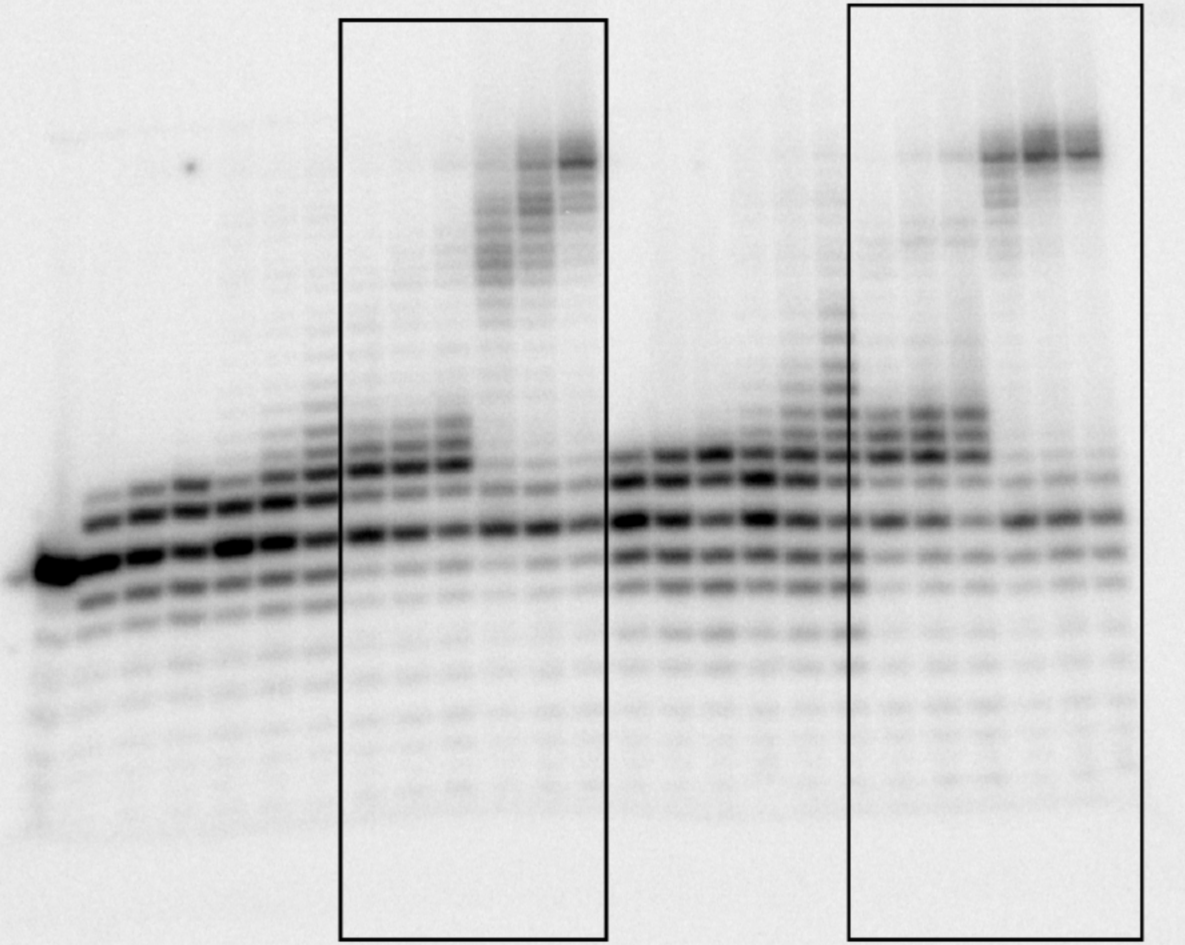

Figure 2A

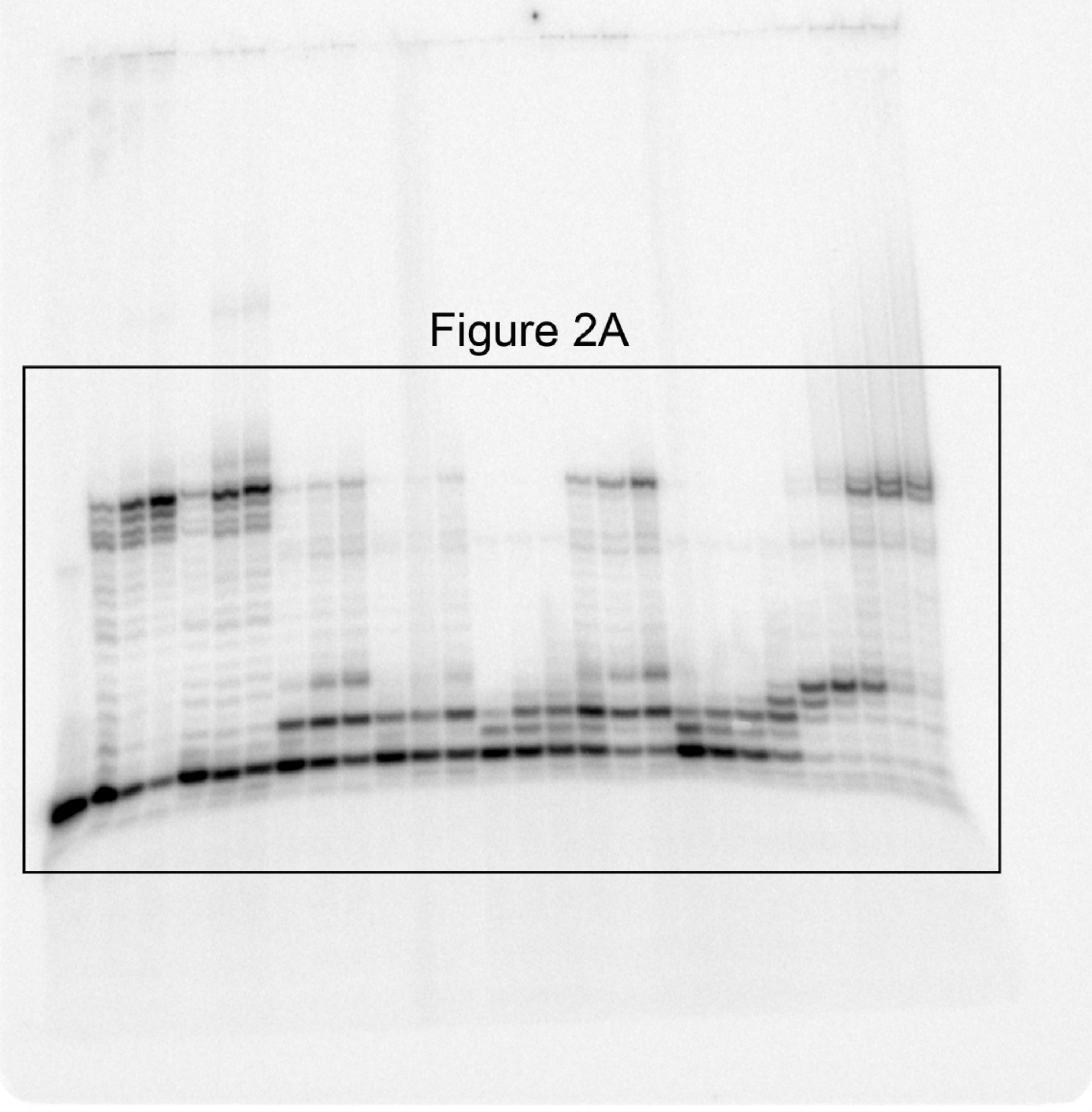

Figure 2B

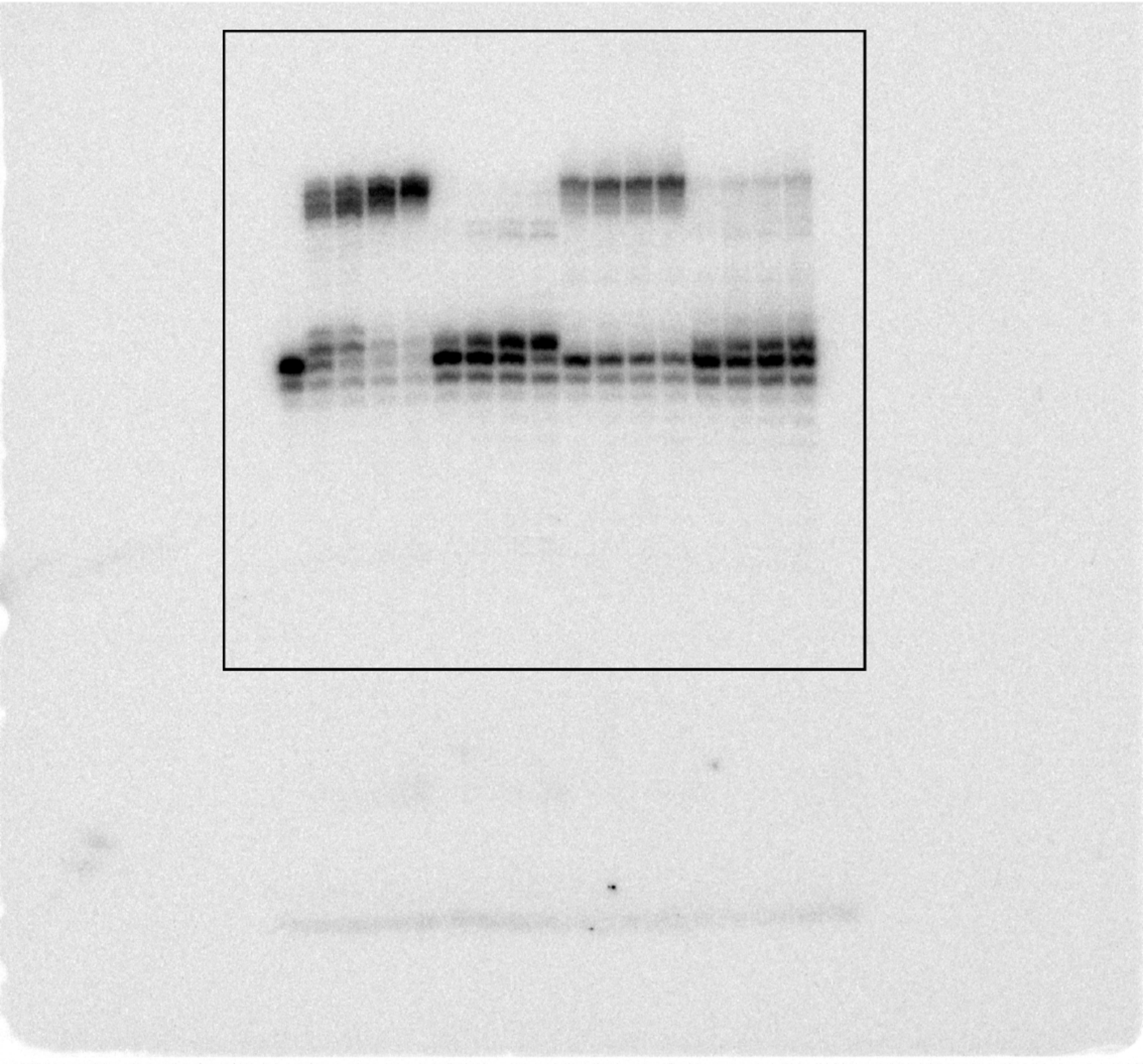

Figure 2B

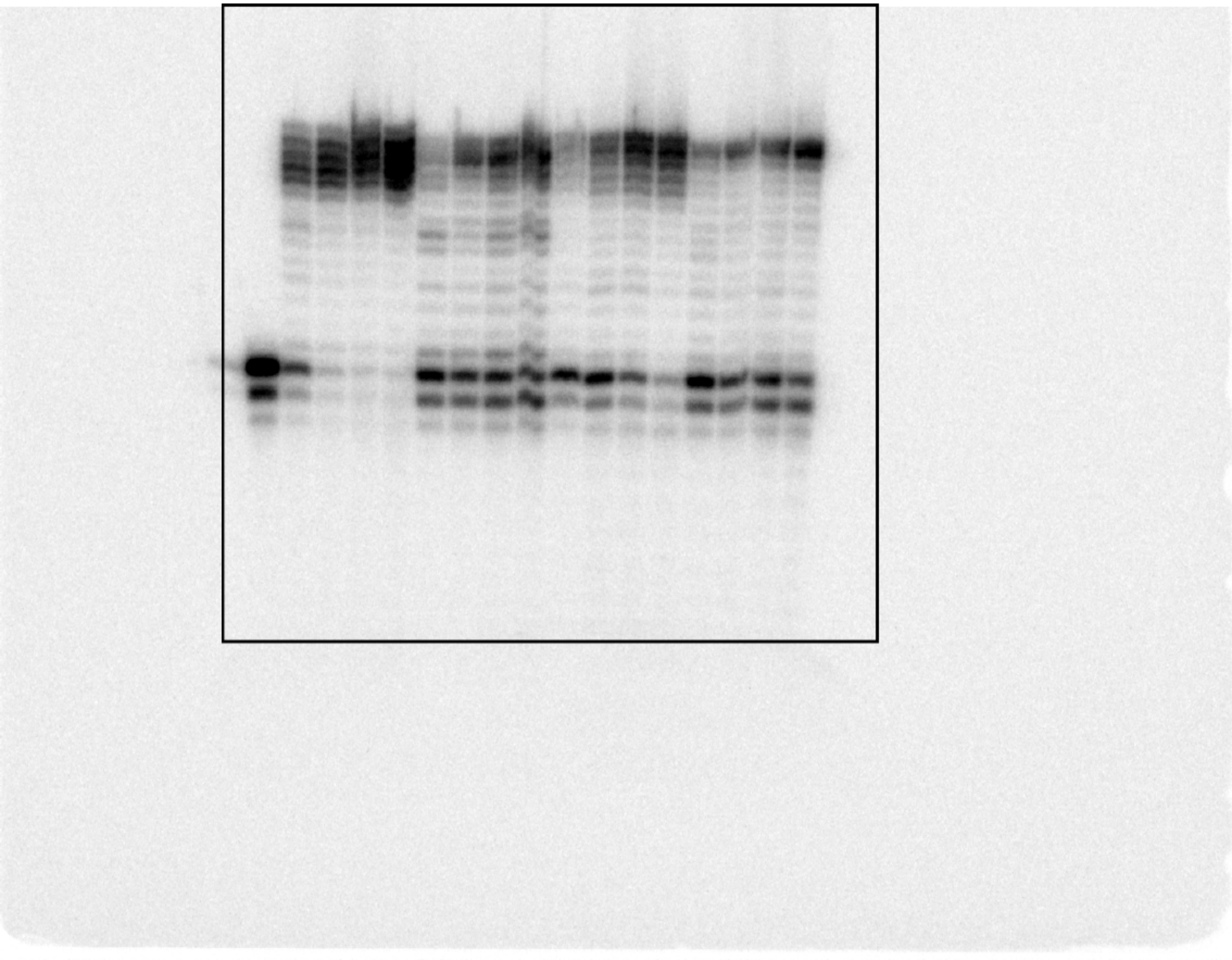

Figure 3A (CisPt CL)

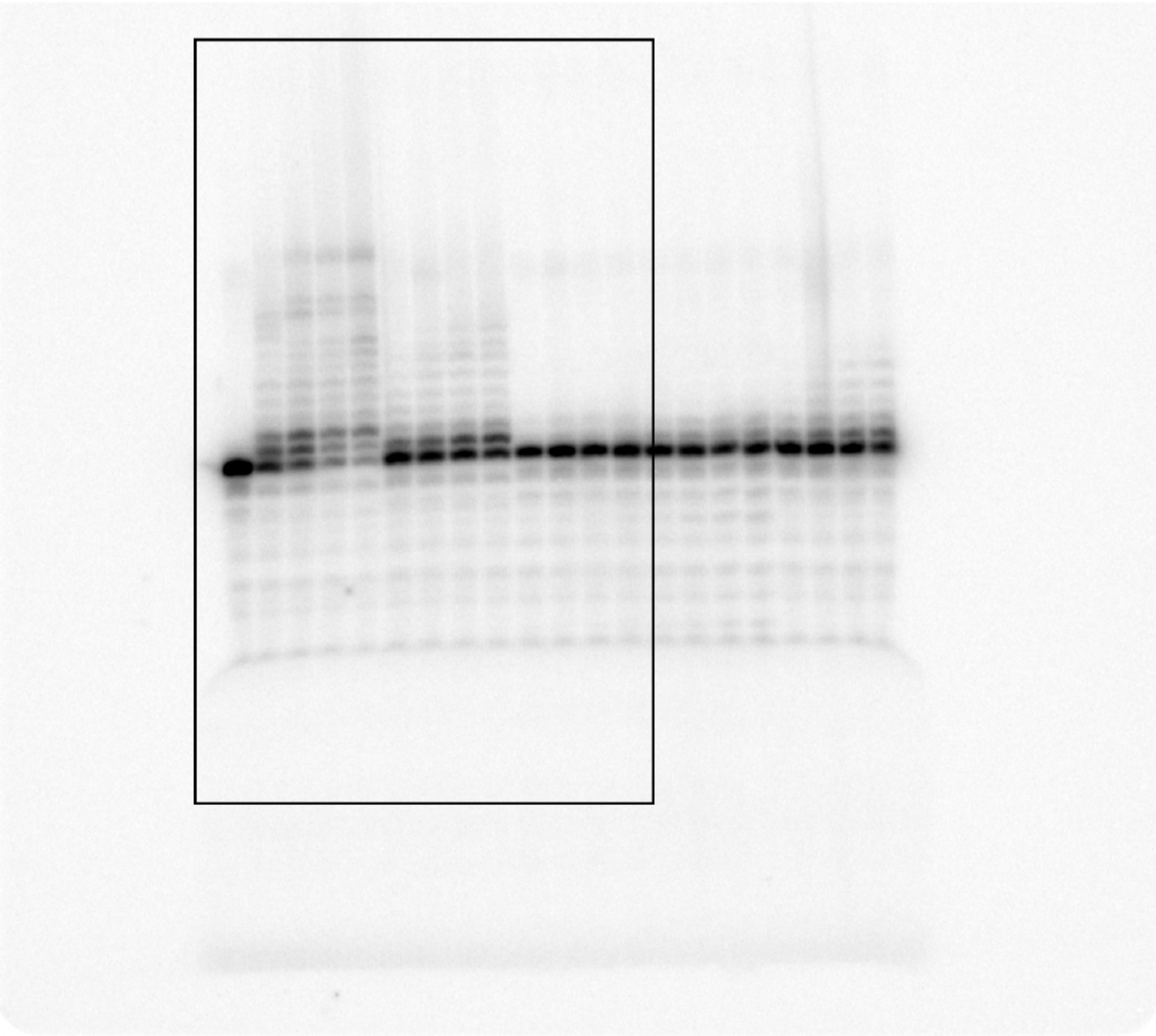

Figure 3A (GG)

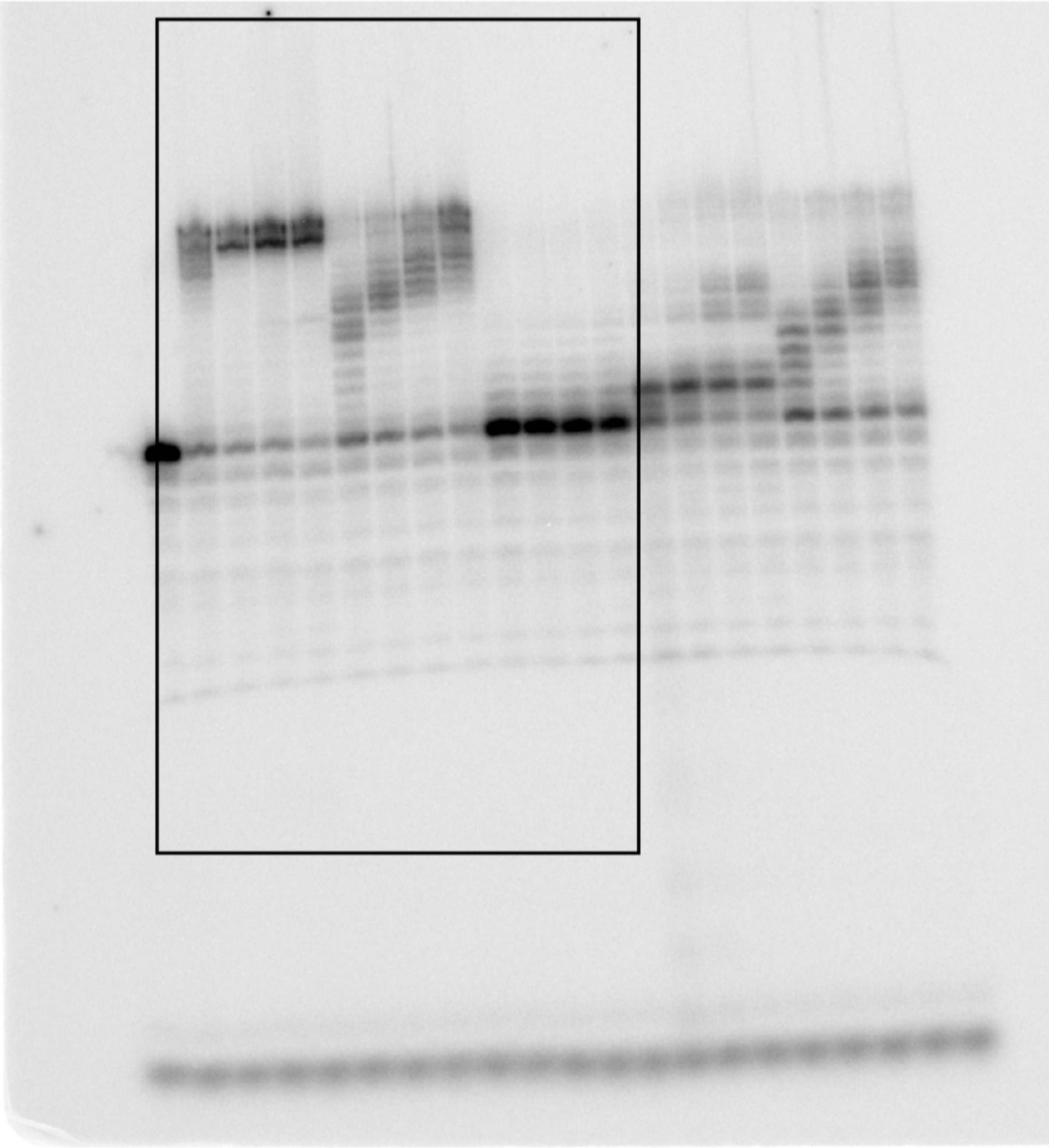

Figure 3B

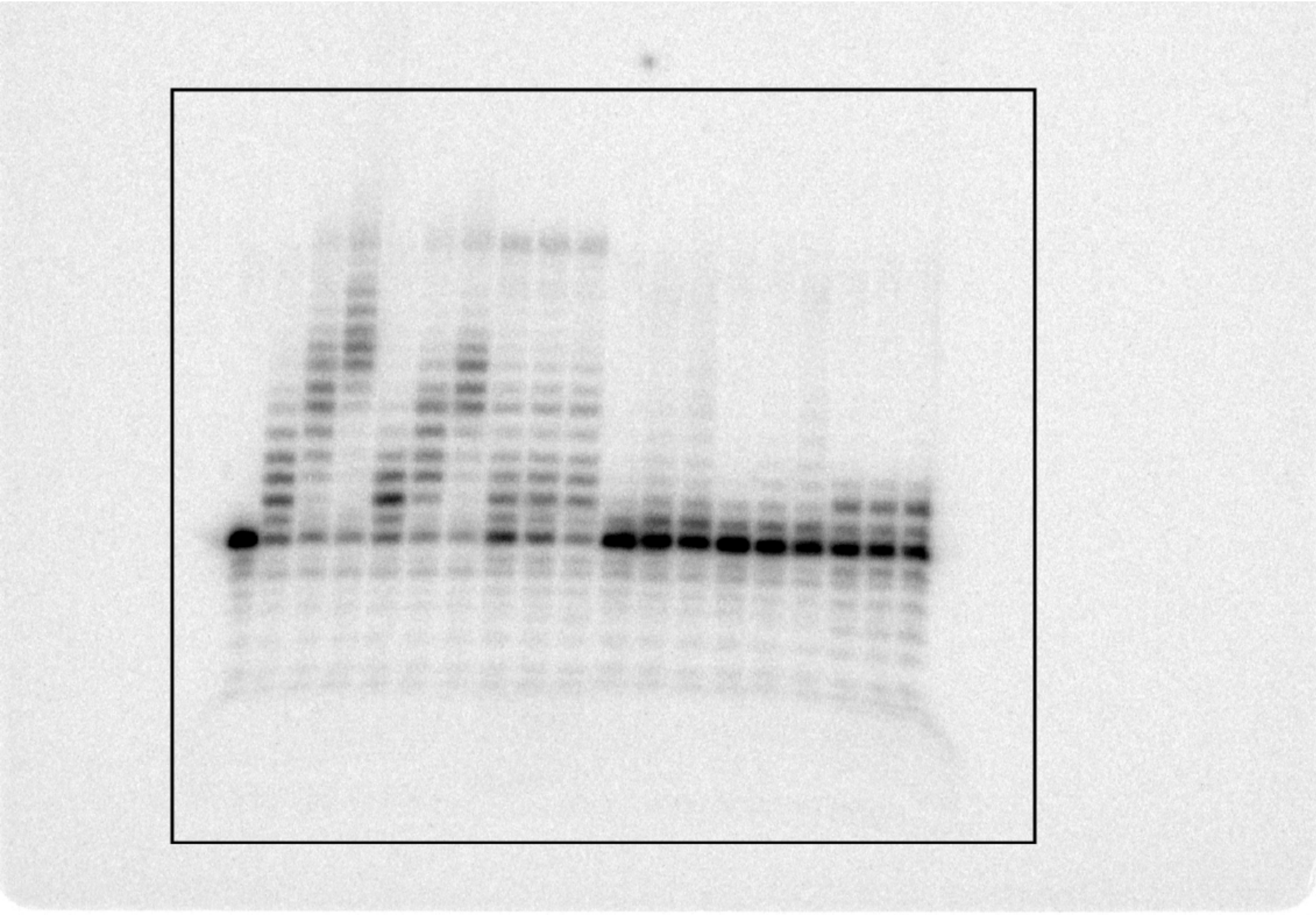

Figure 3C

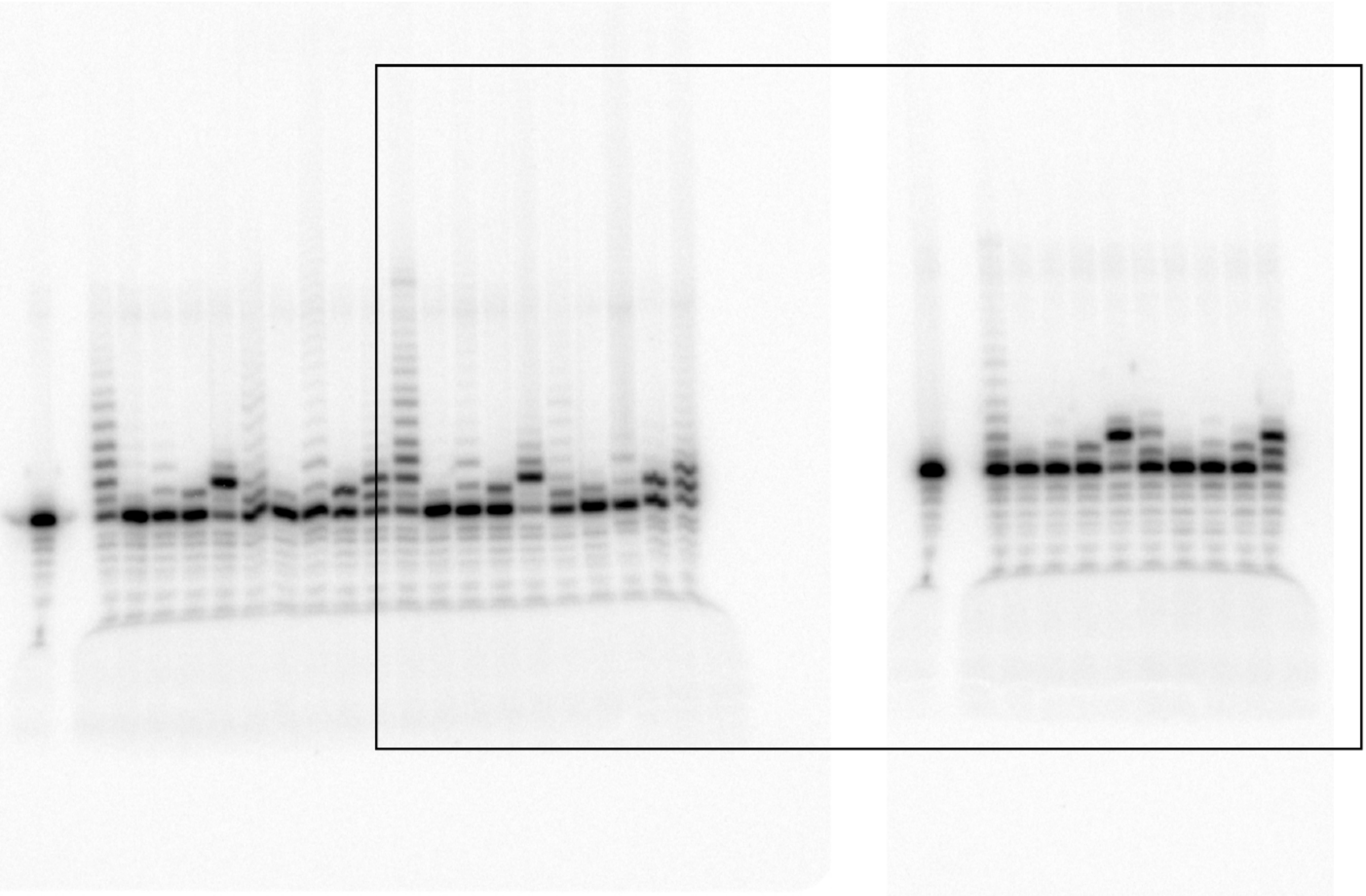

Supp. fig. 1 C

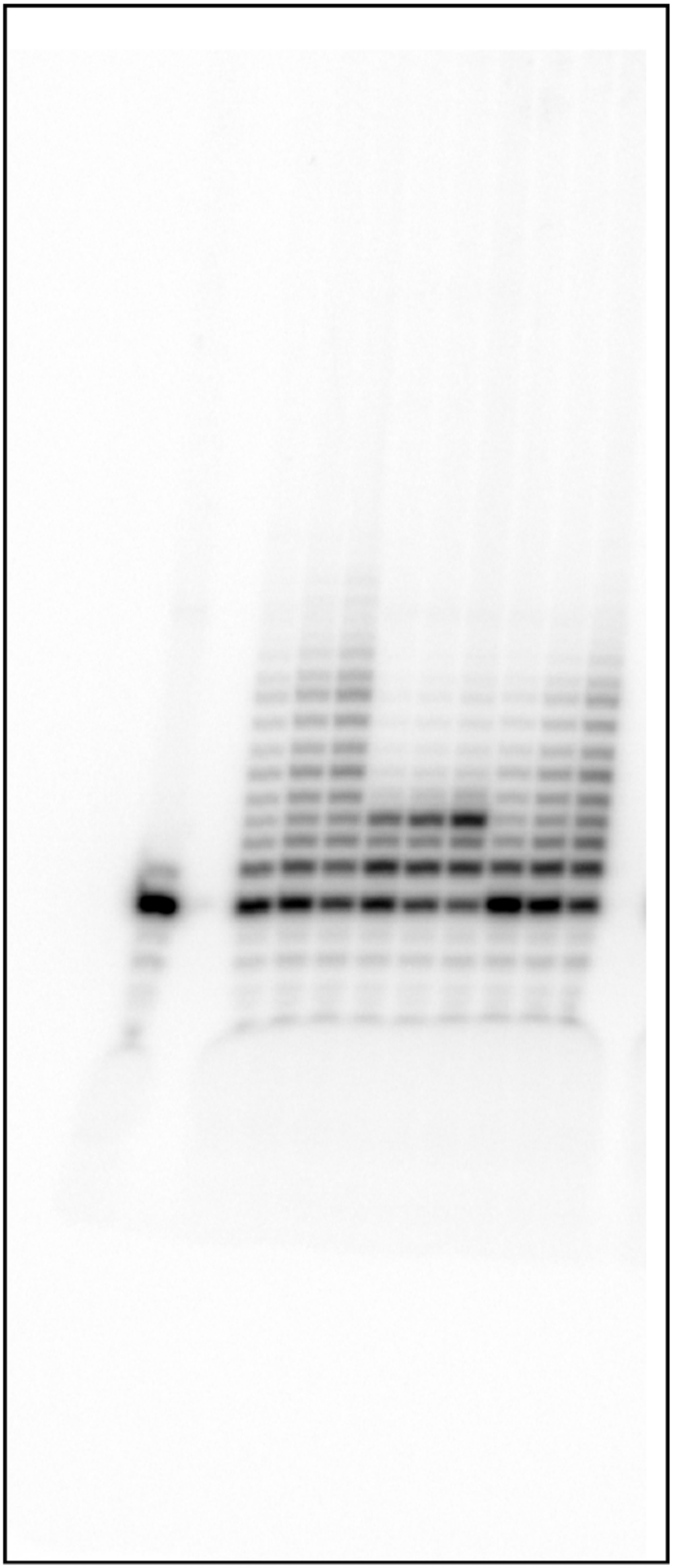

Supp. fig. 1D

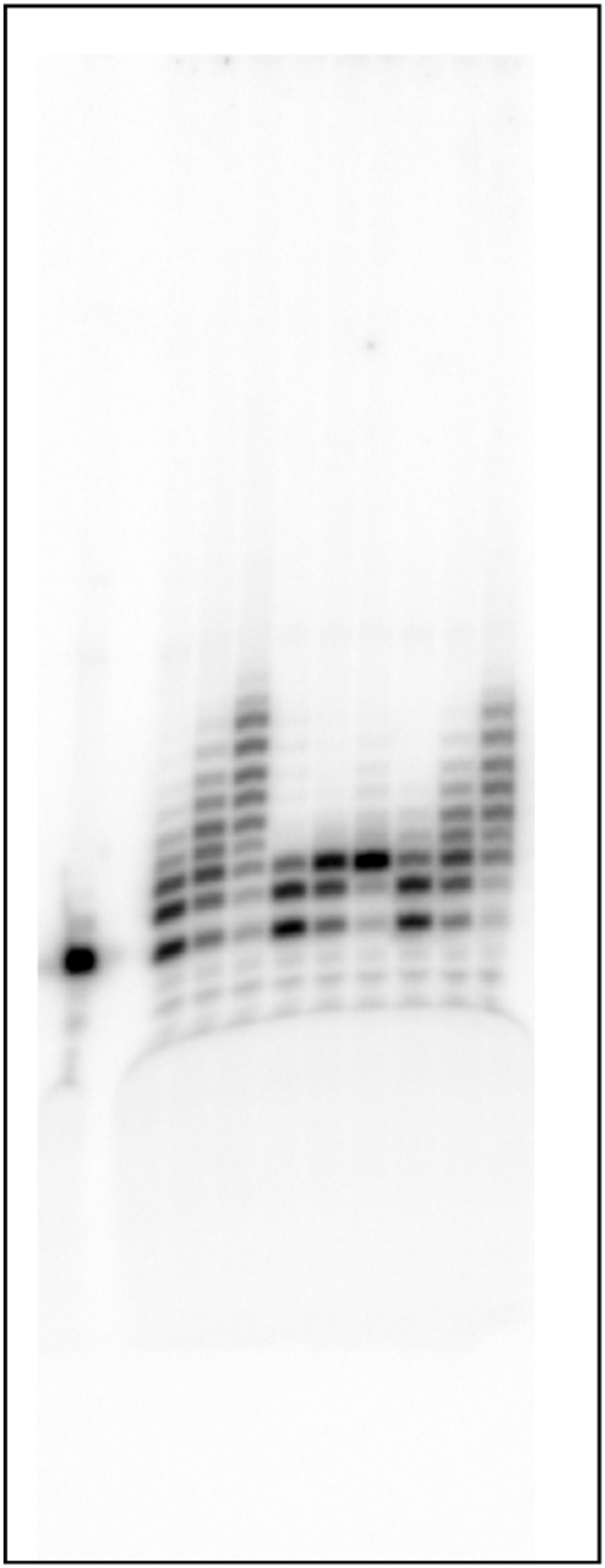

Supp. fig. 1B

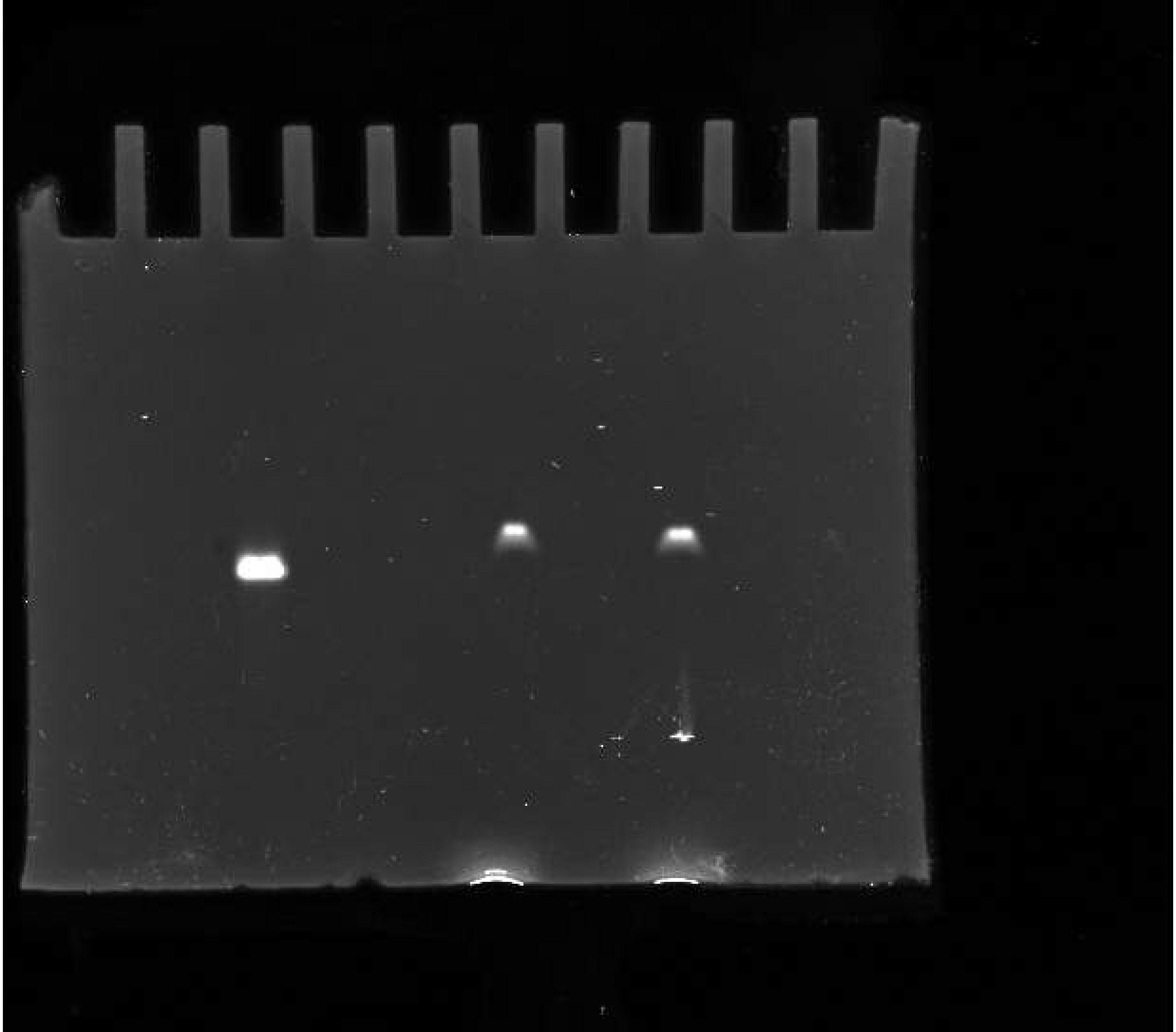

Supp.6A

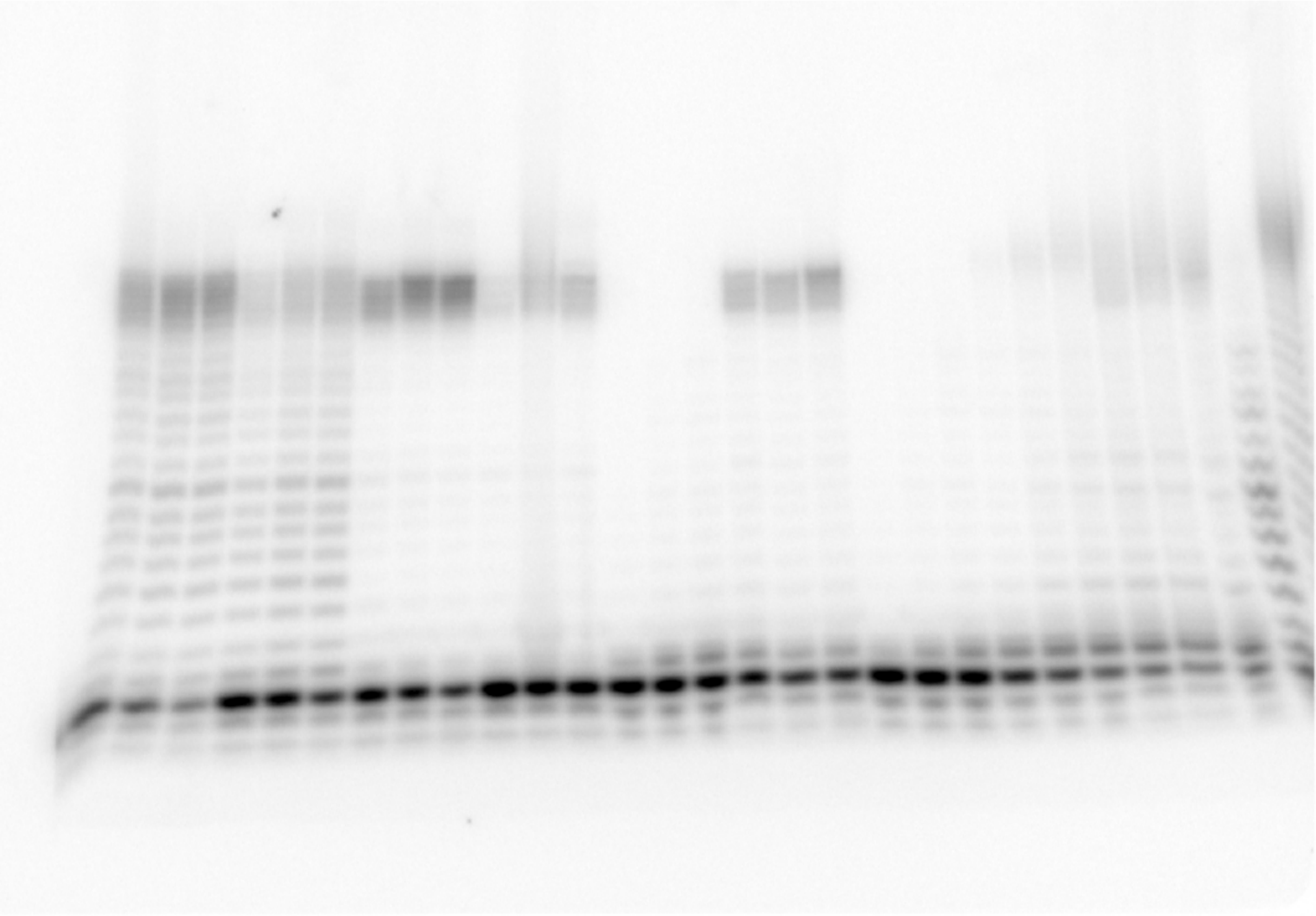

Supp.fig. 2

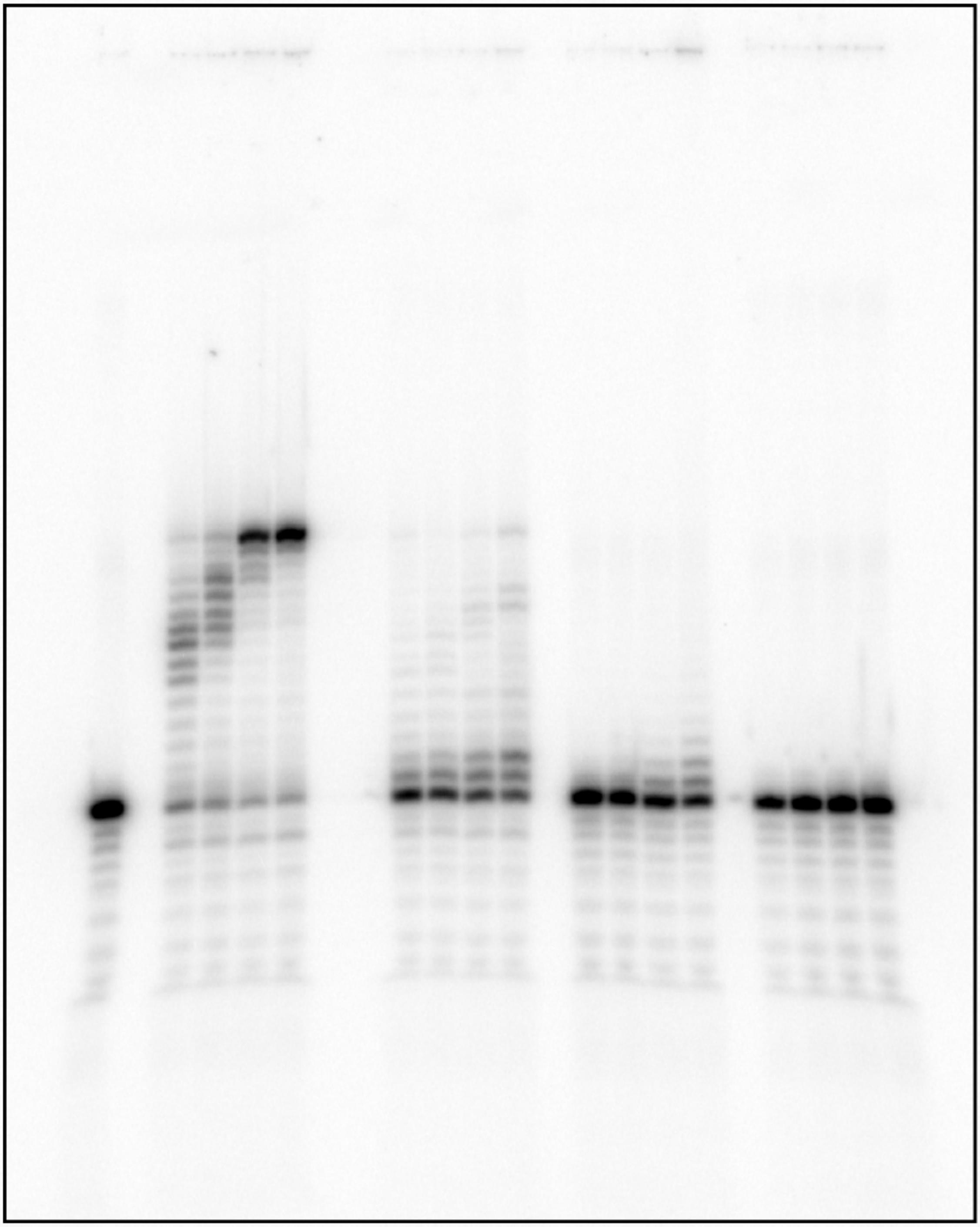

Supp.fig. 3A

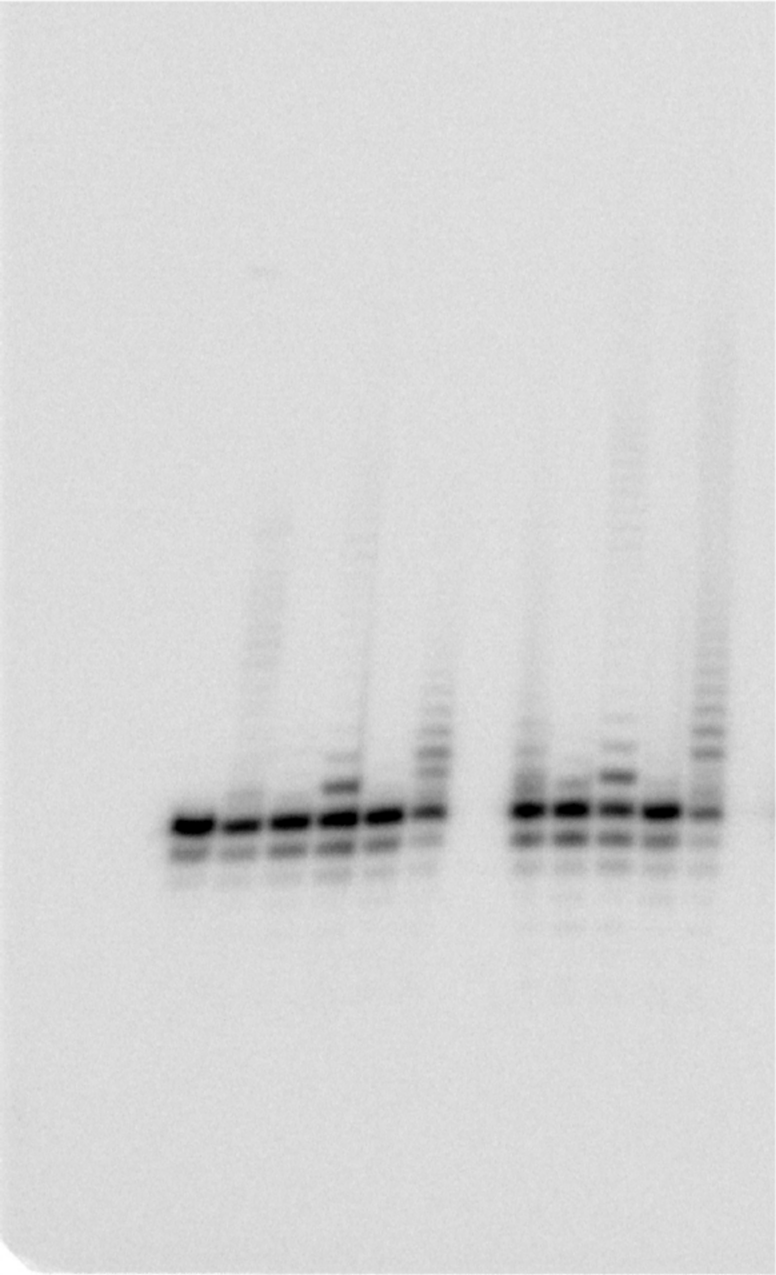

Supp.fig.3B

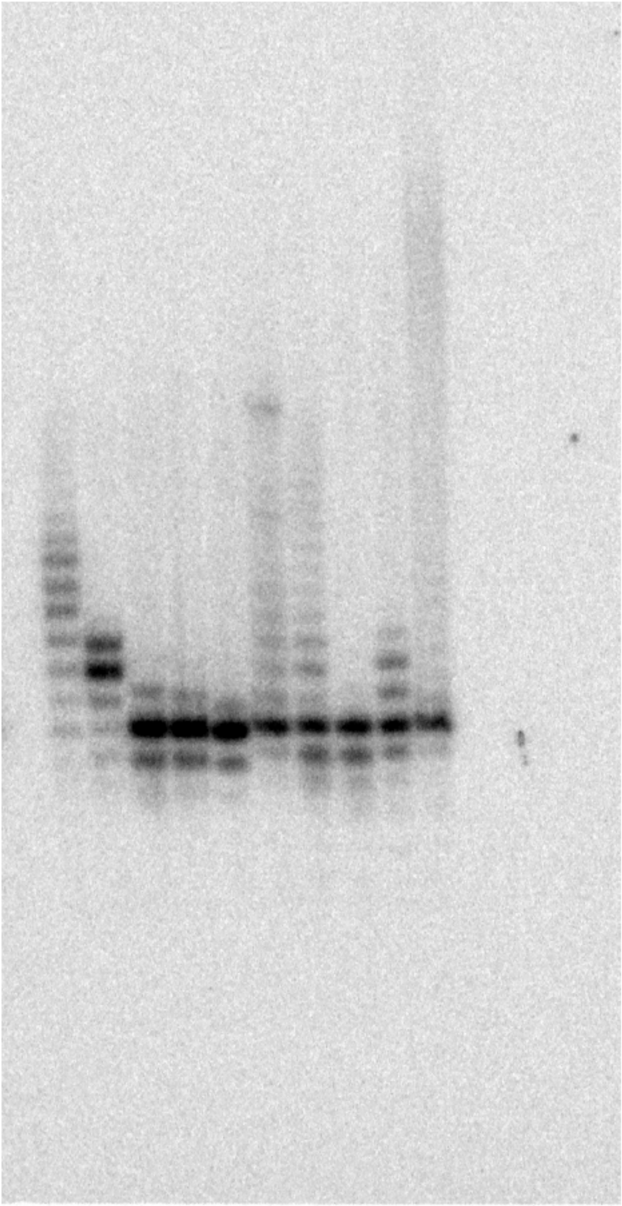

Supp.fig.3C

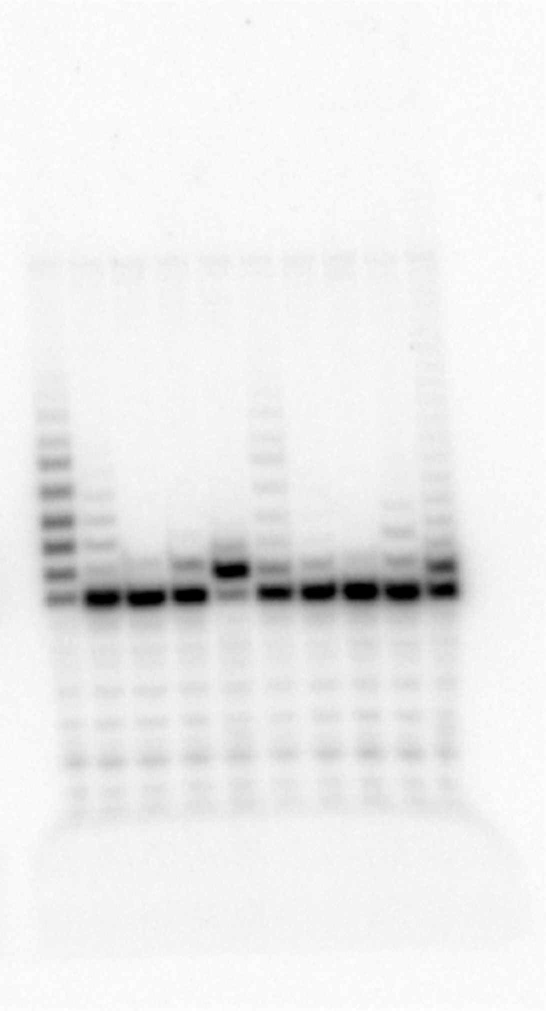

Supp. fig. 3D

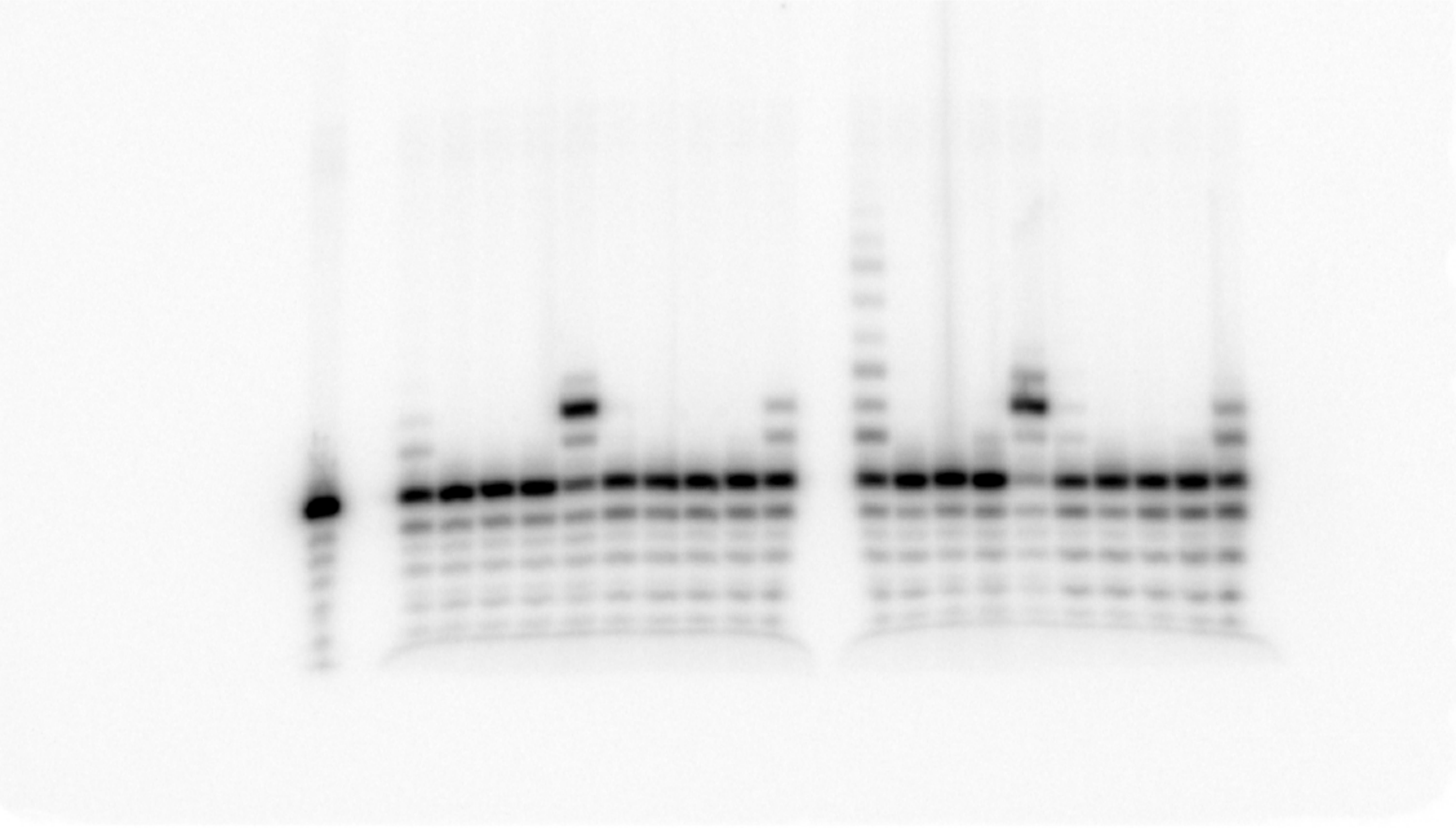

Supp.fig.4

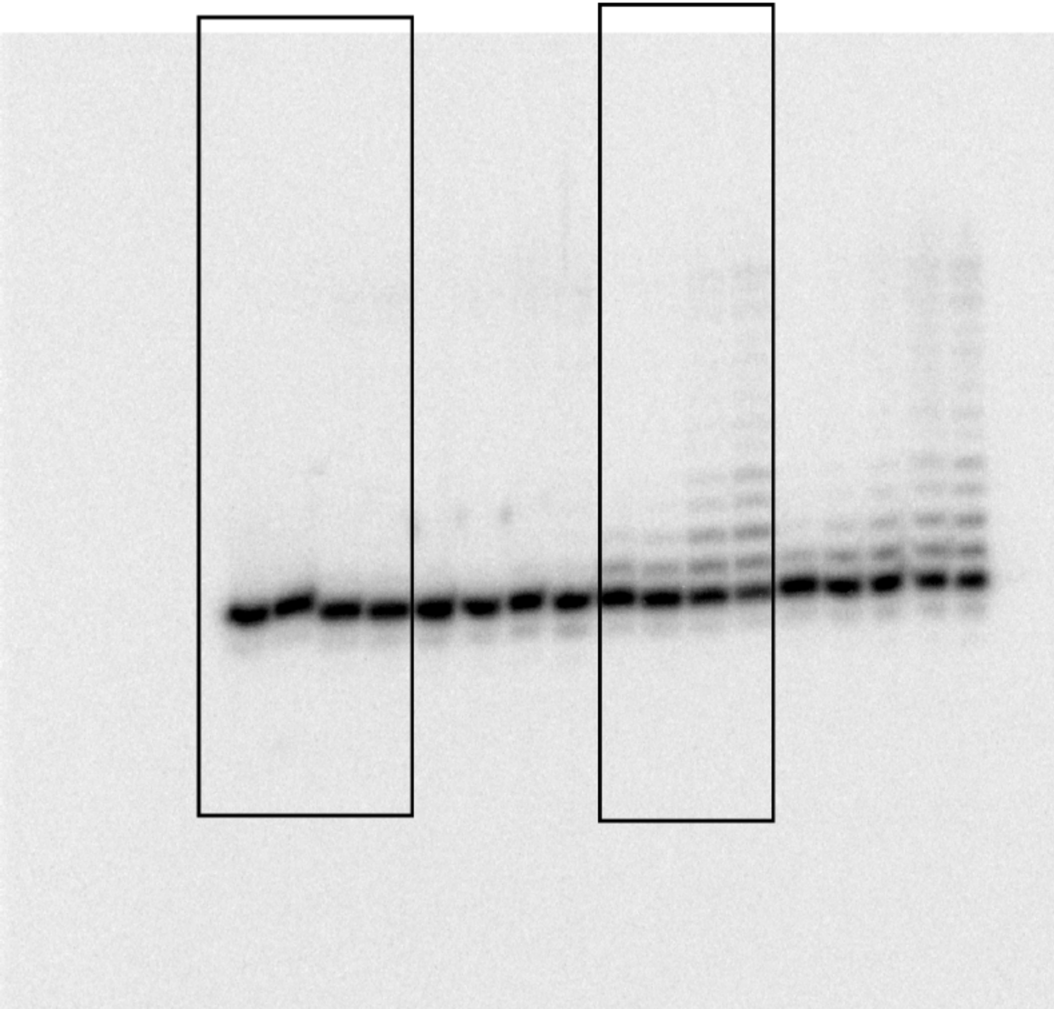

Supp.fig. 5

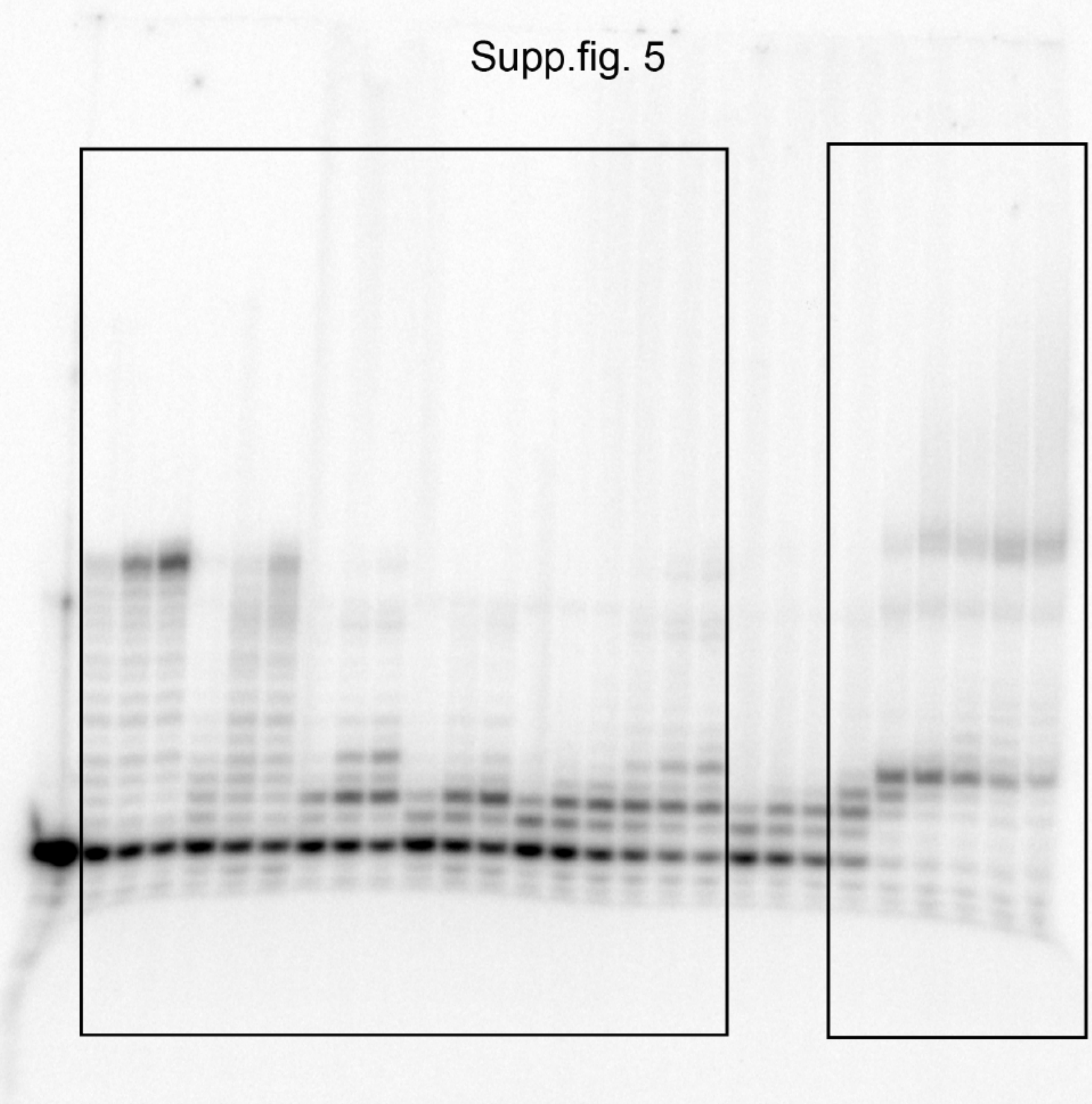

Supp.fig. 6B

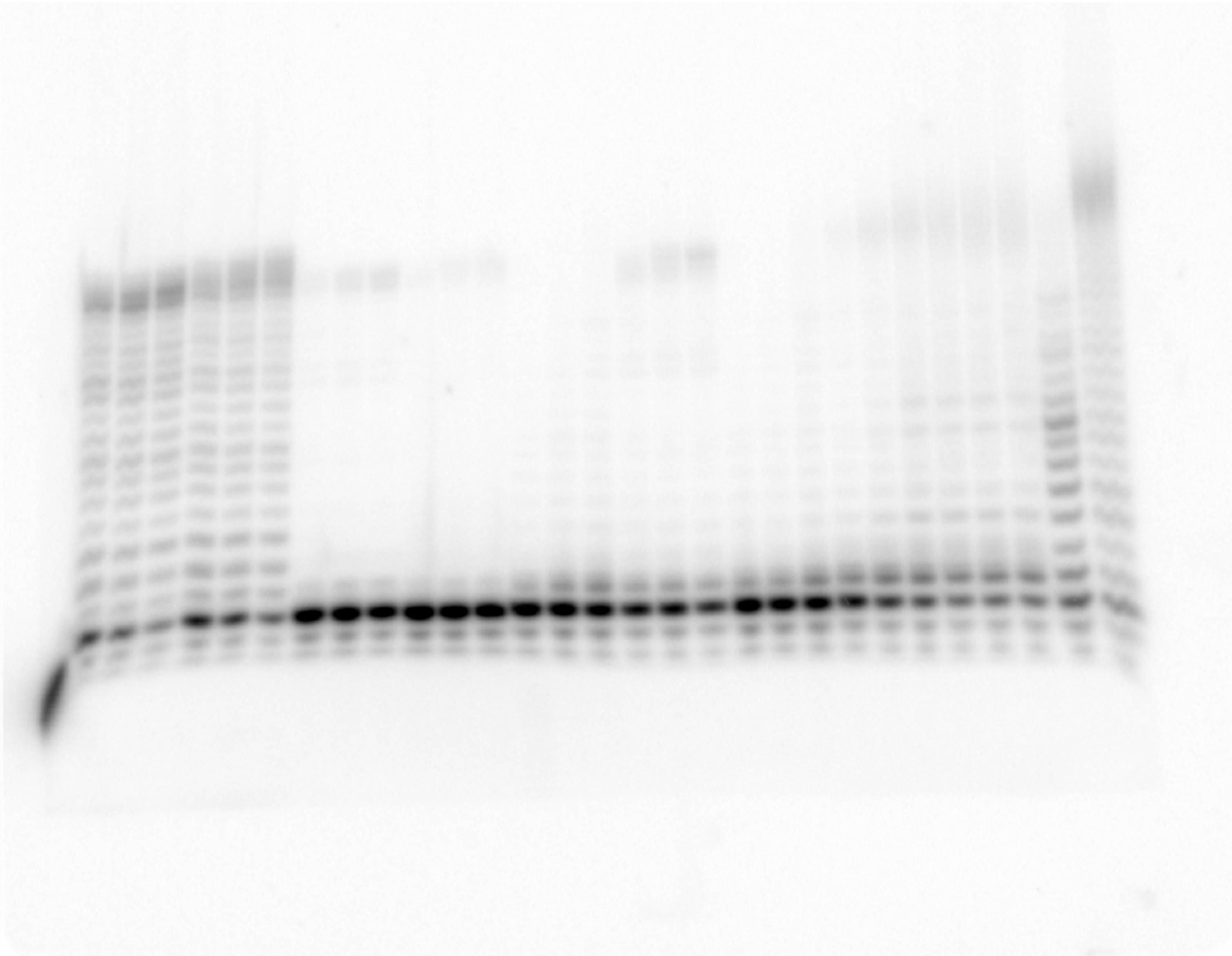

Supp,fig 6C

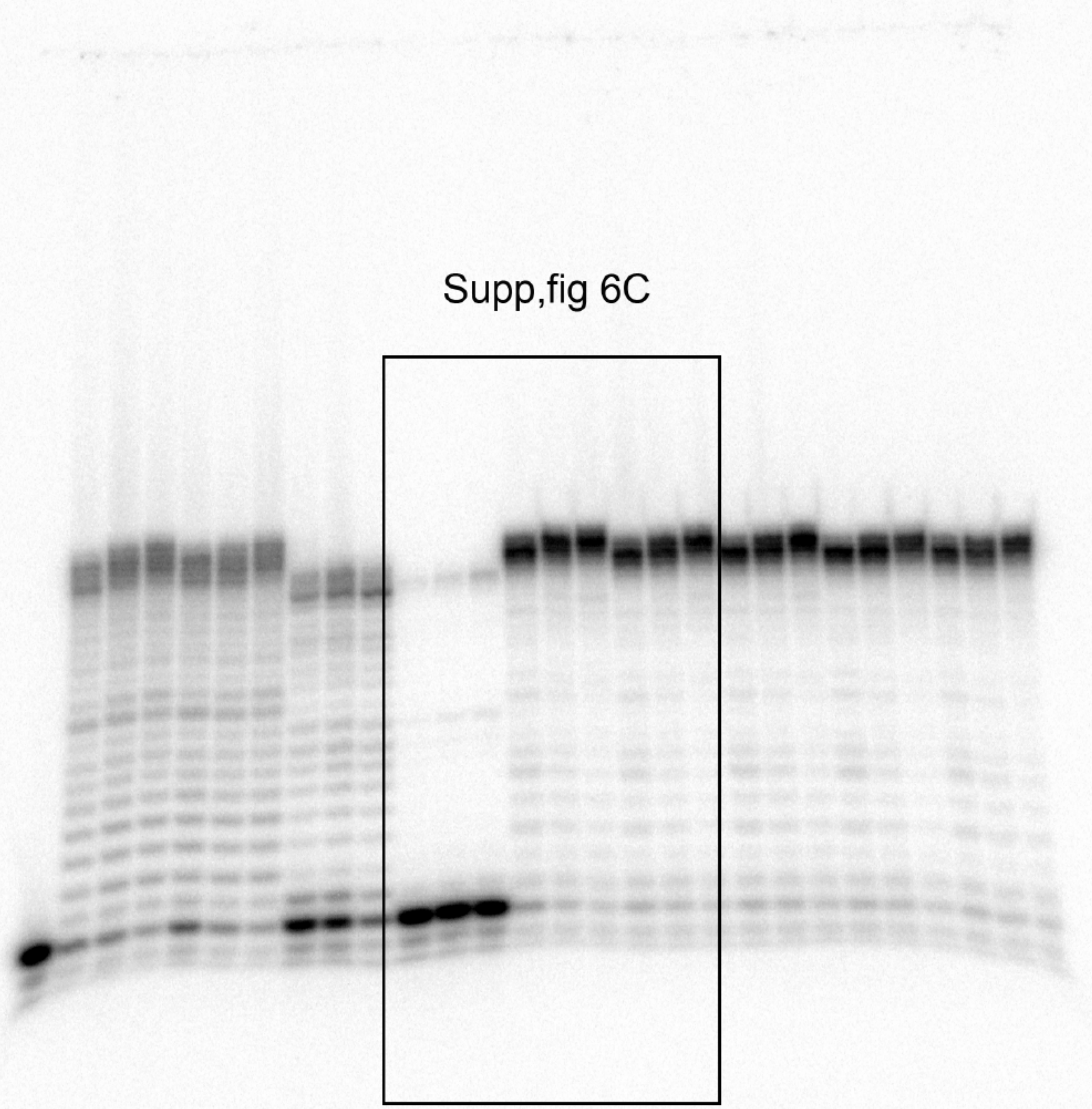

Supplement: Supplementary file 1 — Supplementary Information. [file 41598_2021_96692_MOESM1_ESM.pdf]
